# Temporal changes in per and polyfluoroalkyl substances and their associations with type 2 diabetes

**DOI:** 10.1038/s41598-025-05422-1

**Published:** 2025-07-01

**Authors:** Vivian Berg, Dolley D. Charles, Sandra Huber, Therese H. Nøst, Torkjel M. Sandanger, Maria Averina, Ingvar A. Bergdahl, Mia M. Nilsen, Tom Wilsgaard, Charlotta Rylander

**Affiliations:** 1 Department of Medical Biology, Faculty of Health Sciences, UiT - The Arctic University of Norway, Stakkevollan, P.O Box 6050, 9037 Tromsø, Norway; 2 Department of Laboratory Medicine, University Hospital of North-Norway, Tromsø, Norway; 3 Department of Community Medicine, Faculty of Health Sciences, UiT - The Arctic University of Norway, Tromsø, Norway; 4 Department of Public Health and Nursing, HUNT Research Centre, Norwegian University of Science and Technology, 7491 Trondheim, Norway; 5 The Climate and Environmental Research Institute NILU, Tromsø, Norway; 6 Department of Public Health and Clinical Medicine, Section of Sustainable Health, Umeå University, Umeå, Sweden

**Keywords:** Per- and polyfluoroalkyl substances, Type 2 diabetes mellitus, Temporal change, Prospective case–control study, Repeated measurements, Pre- and post-diagnostic associations, Environmental sciences, Endocrine system and metabolic diseases, Metabolic disorders

## Abstract

We assessed temporal changes of PFAS and associations with T2DM over a period of 30 years in a nested case–control study with repeated measurements. Logistic regression was used to assess associations between 11 PFAS and T2DM at five time-points in 116 cases and 139 controls (3 pre- and 2 post-diagnostic time-points in cases). Mixed linear models were applied to assess if changes in PFAS were related to T2DM status. In the pre-diagnostic time-point T3 (2001), future cases had higher concentrations of PFHpA, PFNA, PFHxS and PFHpS compared to controls. In the post-diagnostic time point T5 (2015/16), PFNA and PFOS were higher in prevalent cases. PFHxS and PFHpS were positively associated with future T2DM at the pre-diagnostic time-point T3, whereas PFTrDA were inversely associated with future T2DM at T1 (1986/87) and prevalent T2DM at T4 (2007/8). Temporal changes in PFAS across the study period showed that cases experienced a greater increase in pre-diagnostic concentrations of PFHpA, PFTrDA, PFHxS and PFOSA, as well as a larger post-diagnostic decrease in PFOSA, compared to controls. This study is the first to show that temporal changes in PFAS are associated with T2DM status for certain PFAS, and associations between PFAS and T2DM vary according to sample year.

## Introduction

Per- and Polyfluoroalkyl substances (PFAS) are widely used in consumer products, and due to their persistency and low degradability in the environment, there is concern regarding their potential toxic effects in humans^1^. Exposure to PFAS has been associated with various diseases, including metabolic disorders involving glucose and insulin homeostasis, and the development type 2 diabetes mellitus (T2DM)^2^. T2DM is characterized by metabolic disturbance that develops over time with hyperglycemia and pre-diabetes preceding the actual diagnosis of T2DM^3^. The onset of the disease process is complex, and the timeline for these developments prior to diagnosis is not well-defined. In addition to the well-known risk factors for T2DM (e.g. sedentary lifestyle, high blood pressure and obesity), endocrine disrupting chemicals like PFAS have been suggested as potential risk factors^4^, as well as the organochlorines (OCs), which we have studied in a previous study using the same study population^5^. As opposed to the fat soluble OCs, the PFAS bind to proteins and the molecular mechanisms by which PFAS may influence metabolic processes could involve the peroxisome proliferator-activated receptor alpha (ppar-α) and gamma (ppar-γ), which are crucial in glucose and lipid metabolism and in inflammation, processes integral to the development of T2DM^6,7^. Conversely, metabolic processes associated with T2DM, such as impaired kidney function, could lead to changes in the concentrations of PFAS in the blood affecting the association with T2DM at different time-points^8^.

A complicating factor in studies investigating PFAS exposure and the risk of T2DM in humans, is temporal patterns of exposure. It has been reported that exposure has decreased throughout the 21th century, primarily due to the voluntary phase-out of PFOS and related compounds starting in the year 2000^9^. Consequently, declines in the concentrations of several PFAS in humans have been observed, while concentrations of some longer-chained PFAS, which are not yet regulated, are either increasing or remaining stable^10^. Additionally, PFAS persists longer and accumulates more rapidly in Arctic regions due to a continued transport of PFAS via sea and wind currents to this area, which may result in delayed effects of legislative actions towards PFAS and prolonged exposure duration in Arctic populations.

Given the gradual progression of T2DM and the historical shifts in human exposure to PFAS, a single measurement of PFAS may not sufficiently capture its association with T2DM. As such, health implications may be influenced not only by the current concentration in blood, but likely also by the maximum exposure level attained, the duration of internal exposure, and the temporal pattern of exposure. Recent reviews of studies examining PFAS and T2DM, published in 2022 and 2023^2,11^ have reported inconsistent findings, with positive, negative, and null associations observed between various PFAS and diabetes markers, and T2DM status. The design of these studies, many of which are cross-sectional and include, may partly account for the inconsistent results. To date, only our previous study in women from the NOWAC study has reported measurements of PFAS both prior to and following T2DM diagnosis^12^. To clarify the temporality of associations between PFAS and T2DM, longitudinal studies with multiple repeated measurements in prospective T2DM cases and controls are necessary. Therefore, this study, with up to five repeated measurements in the same individuals over a 30-year period (1986–2016), aimed to compare pre- and post-diagnostic concentrations in T2DM cases relative to controls. The unique study design allowed us to assess associations between PFAS and T2DM, if associations differ over time in future and prevalent cases and for specific compounds, and if changes in PFAS concentrations were influenced by T2DM status.

## Materials and methods

### The Tromsø Study—study design and participants

**Fig. 1:**
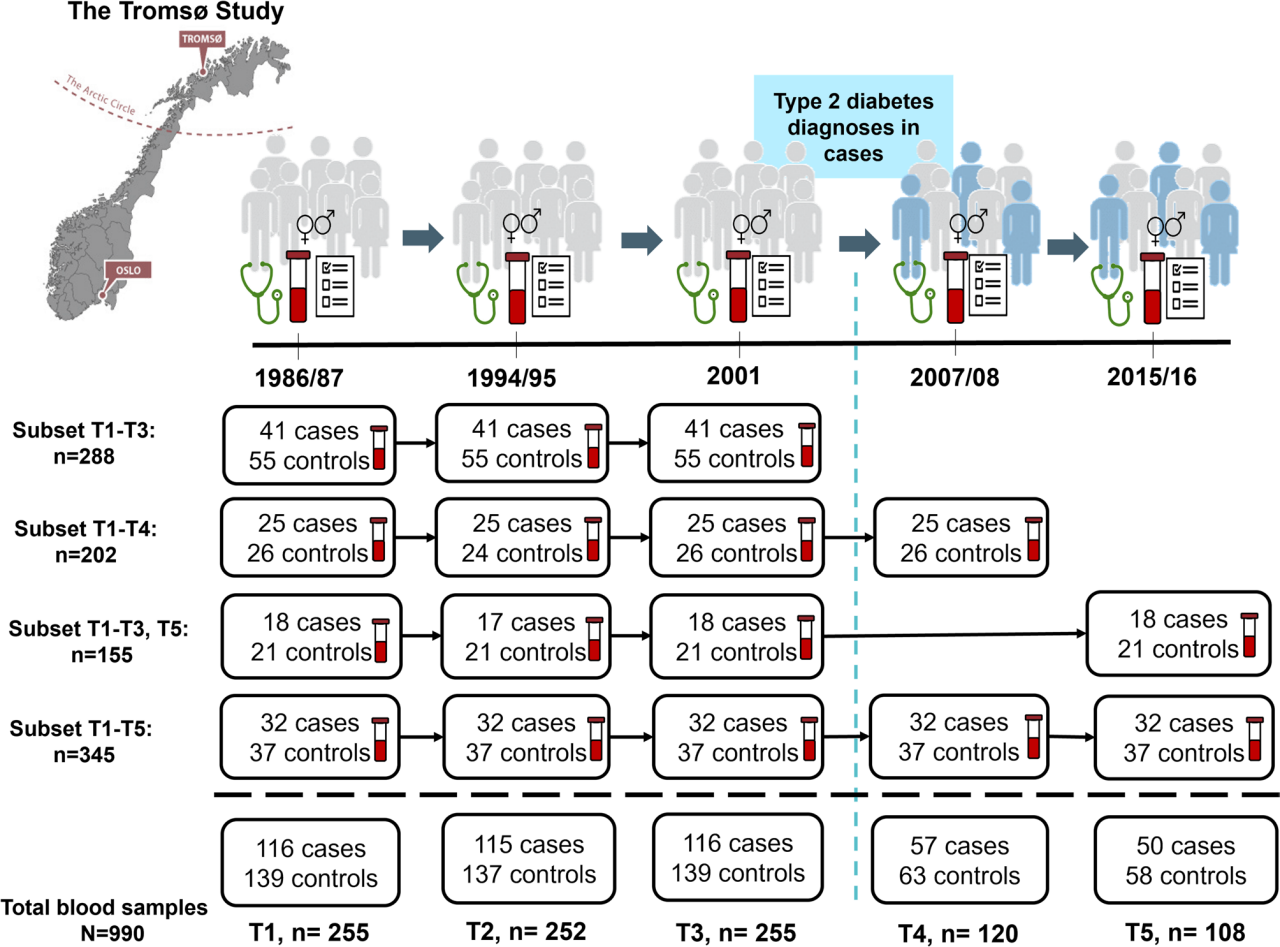
Study overview, sample size, and sample subsets (mutually exclusive groups) according to participation in the different surveys (time-points, T) of The Tromsø Study (1986–2016).

The study was approved by the Regional Committee for Medical Research Ethics and all methods were performed in accordance with the relevant guidelines and regulations. Our study sample is the same as described by Charles et al.^5,13^ and the study is a nested case–control study utilizing a sample drawn from participants of The Tromsø Study, which is a prospective cohort study involving repeated health surveys among residents of Tromsø, a city in Northern Norway. The Tromsø Study is described in detail elsewhere^14^. Briefly, over 15,000 participants have participated in three or more surveys, and our study encompasses individuals who took part in up to 5 surveys, and in at least the three surveys conducted in 1986/87 (Tromsø3), 1994/95 (Tromsø4), and 2001 (Tromsø5), which are subsequently referred to as time-points 1, 2, and 3 (T1, T2, T3) (Fig. 1). Several of these subjects also participated in the 2007/08 (Tromsø6) and 2015/16 (Tromsø7) surveys, denoted as T4 and T5. Initially, 76 women and 69 men fulfilled the inclusion criteria of having a diabetes diagnosis registered in a local diabetes registry between T3 and T4, and had available frozen pre-diagnostic serum samples from T1, T2 and T3 according to the Tromsø study data registry. 76 women and 69 men without a T2DM diagnosis and available frozen serum samples at the same time-points, were randomly selected as controls. We did not match on age as we believed the inclusion criteria of having participated in at least the three surveys T1, T2 and T3 would result in approximate age for cases and controls. T2DM diagnoses were verified in blood samples by HbA1c measured and recorded at each survey, and we excluded twenty-nine cases that had HbA1c ≥ 6.5% in pre-diagnostic samples, and five controls had HbA1c ≥ 6.5% at one of the time-points. This resulted in a maximum number of cases and controls at any one time-point being 116 and 139, respectively (at T1 and T3), with four subsets (mutually exclusive groups) with 255 participants at T1 and T3, 252 at T2 (three participants (1 case and 2 controls) did not have enough serum left for analysis at T2), 120 at T4, and 108 at T5 adding up to total of 990 samples (Fig. 1). In addition to providing blood samples at each survey, participants underwent clinical examinations, and completed questionnaires about their lifestyle, including medication use, parity, breastfeeding (information about breastfeeding was not recorded at T1), education and physical activity at all surveys.

### Chemical analyses

Concentrations of total cholesterol, high-density lipoprotein (HDL) cholesterol, low-density lipoprotein (LDL) cholesterol and triglycerides, were performed on a Cobas® 8000 platform (Roche Diagnostics, Rotkreuz, Switzerland) by laboratory staff at Department of Laboratory Medicine, University Hospital of North Norway. Total lipids were calculated according to the formula proposed by Philips et al.: Total lipids = 2.27*total cholesterol + triglycerides + 0.623 (g/L)^15^.

A panel of 24 PFAS were determined in thawed serum samples at the Environmental Pollutant Laboratory, Department of Laboratory Medicine, including the following PFAS: Perfluorobutanoic acid (PFBA), perfluoropentanoic acid (PFPA), perfluorohexanoic acid (PFHxA), perfluoroheptanoic acid (PFHpA), perfluorooctanoic acid (PFOA), pefluorononanoic acid (PFNA), perfluorodecanoic acid (PFDA), perfluoroundecanoic (PFUnDA), perfluorododecanoic adic (PFDoDA), perfluorotridecanoic acid (PFTrDA), perfluorotetradecanoic acid (PFTeDA perfluorobutane sulfonic acid (PFBS), perfluoropentane sulfonic acid (PFPS), perfluorohexane sulfonic acid (PFHxS), perfluoroheptane sulfonic acid (PFHpS), perfluorooctane sulfonic acid (PFOS), perfluorononane sulfonic acid (PFNS), perfluorodecane sulfonic acid (PFDS), perfluorododecane sulfonic acid (PFDoDS), 4:2 fluorotelomer sulfonic acid (4:2 FTSA), 6:2 fluorotelomer sulfonic acid (6:2 FTSA), 8:2 fluorotelomer sulfonic acid (8:2 FTSA), 10:2 fluorotelomer sulfonic acids (10:2 FTSA) and perfluorooctane sulfonamide (PFOSA). Procedures for instrumental analysis, sample preparation, quantification, and quality control has been previously described in detail^16^. In short, sample preparation of 50 µl of serum was conducted on a liquid handler workstation (Tecan, Männedorf, Switzerland) and by solid-phase micro-elution in 96-well format. The extract was separated by reversed phase liquid chromatography on an Acquity UPLC HSS 3 T column (2.1 × 100 mm, 1,8 µm; Waters, Milford, MA, USA). PFAS molecules were ionized by negative electrospray and recorded by the Xevo-TQ-S tandem-mass-spectrometer in multiple-reaction-mode (Waters, Milford, MA, USA). Masslynx and Targetlynx software (Version 4.1, Waters, Milford, MA, USA) was used for quantification and attained by the internal-standard method with isotope-labelled PFASs. The linear isomers of perfluorosulfonic acids (PFSA) were used for calculating the contribution of the branched compounds. Concentrations of PFSA are reported as sum concentrations of branched and linear isomers. For each individual sample, the Targetlynx software calculated concentrations for method detection limits (MDLi) and each individual PFAS with a signal to noise ratio of three divided by the respective sample amount. Blank samples (n = 4), SRM 1957 and SRM 1958 (NIST, Gaithersburg, MD, USA) and bovine serum samples (n = 3) (Sigma Aldrich, Steinheim, Germany) were prepared and analyzed within each batch of 96 samples for quality control. SRM samples were within the acceptance limits with correlation coefficients of variation < 13% for measured PFAS. All PFAS analyses were within the acceptable ranges of the international quality control program, “the Arctic Monitoring and Assessment (AMAP) Ring Test for Persistent Organic Pollutants in Human Serum” organized by the Laboratory of toxicology (Laboratoire de toxicologie, Institut National de Santé Publique du Quebec, Canada).

Compounds with detection frequencies above 70% (Supplemental Table S1) were included in the statistical analyses and concentrations below the sample-specific MDLs were replaced by MDL divided by the square root of 2.

### Statistical analyses

Concentrations of PFAS at each time-point for both cases and controls are presented as means, medians and minimum and maximum in tables in the supplemental material. Box plots are also used to graphically illustrate the means, medians, and percentiles. We report the mean differences and 95% confidence intervals (CIs) for participant characteristics with parametric distributions, as well as the p-value from the Mann–Whitney test for differences in PFAS concentrations (non-parametric distributions) between cases and controls at each time-point.

To assess associations between PFAS concentrations and T2DM at each time-point we used logistic regression models with the different PFAS as continuous independent variables and T2DM status as dependent variable. With our study design this means that at time-points T1, T2 and T3 we assess the association between PFAS concentrations and odds of developing T2DM, whereas for T4 and T4 we assess the odds of having T2DM according to concentrations of PFAS. These results are reported as odds ratios (ORs) per one quartile increase in PFAS concentration along with 95% CIs. Additionally, we conducted the same models with PFAS grouped as summed perfluoroalkyl carboxylic acids (ΣPFCA [PFHpA + PFOA + PFNA + PFDA + PFUnDA + PFDoDA + PFTrDA]) and sulfonates ΣPFSA (PFHxS + PFHpS + PFOS + PFOSA). To identify potential confounding factors in the causal pathway between PFAS and T2DM, we constructed a directed acyclic graph (DAG) based on the existing literature including the potential casual factors: education level, diet, parity, breastfeeding, sex, age, BMI, weight change, total lipids and physical activity. According to the DAG (Supplemental Figure S1), the confounders to be included in our analysis were sex, age (in years), BMI (kg/m^2^), weight change (in kilograms), parity, breastfeeding (total months), physical activity (categorized into active/inactive), and education level (category 1–5, lowest to highest education level completed). Total lipids are treated as mediators and could not be adjusted for. Information on weight change was calculated using weight information from two consecutive time-points (for example: weight change at T3 = [weight at T3] − [weight at T2]). Information on parity, breastfeeding, physical activity and educational level was extracted from questionnaires. Diet was treated as unobserved, as we did not have detailed information on dietary habits. Weight change at T1 was set to zero, as we lacked weight information from the preceding Tromsø survey. Breastfeeding duration at each time-point was determined by summing the total number of months of breastfeeding for each child.

To assess time trends in PFAS from T1–T3 and T3–T5 (including all time points and with T3 as reference) in cases and controls, while accounting for dependencies between repeated measurements, linear mixed models were used including a random intercept for individuals and an unstructured variance covariance matrix for the residual for the repeated measurements^17^. Log-transformed PFAS concentrations were included as continuous dependent variables, whereas the independent variables were T2DM status and sex (constant over time), and the time-indicator variables of each survey, weight change, BMI (normal: ≤ 24.9 kg/m^2^, overweight: ≥ 25.0 to ≤ 29.9 kg/m^2^, obese: ≥ 30 kg/m^2^), parity, breastfeeding, physical activity and educational level (time dependent). We included interaction terms between T2DM status and time. Estimated PFAS concentrations from the linear mixed models were plotted for T2DM cases and controls at each time-point. To assess if the associations between PFAS concentrations and T2DM status differed by sex or age group, we conducted stratified analyses. Separate models were fitted for males and females, as well as for age groups (17–44, 45–50, 51–61 years at T1). To control for false positive findings due to multiple comparisons, we reported the 99.5% CIs, which corresponds to a Bonferroni correction for 10 statistical tests and a significant level of 0.005. STATA software, version 17 (StataCorp, 4905 Lakeway Drive, College Station, TX, USA) were used for all statistical analyses.

## Results

### Study population characteristics

The study sample consisted of 54% and 52% female cases and controls, respectively. The cases were, on average, slightly older than the controls by approximately 2.5 years, had higher concentrations of total lipids at all the pre-diagnostic time-points, and had a higher body weight (with a mean difference of 7.5–10 kg across the different time-points) and a higher BMI at all time-points (mean difference ranging from 3.2 to 4.1 kg/m^2^) (Table 1). Parity, the duration of breastfeeding, physical activity, and education level were comparable between cases and controls except for that the controls were more physical active at T2 and T5 compared to the cases.

**Table 1:** Participant characteristics are displayed as means and standard deviations (SD) for cases and controls, along with the mean difference (ΔMean) between type 2 diabetes mellitus cases and controls at each pre- and post-diagnostic time point (T) in The Tromsø Study (1986–2016).

Characteristics		Pre-diagnostic time-points						Post-diagnostic time-points			
		T1 (1986/87)		T2 (1994/95)		T3 (2001)		T4 (2007/08)		T5 (2015/16)	
		Mean ± SD	ΔMean (95% CI)	Mean ± SD	ΔMean (95% CI)	Mean ± SD	ΔMean (95% CI)	Mean ± SD	ΔMean (95% CI)	Mean ± SD	ΔMean (95% CI)
Age (years)	Cases Controls	47.5 ± 7.63 45.0 ± 9.85	2.49 (0.28, 4.69)	55.5 ± 7.65 53.0 ± 9.90	2.48 (0.25, 4.71)	62.5 ± 7.63 60.0 ± 9.85	2.49 (0.28, 4.69)	65.9 ± 7.38 63.4 ± 9.44	2.58 (0.50, 5.67)	73.6 ± 7.02 69.9 ± 10.4	3.72 (0.27, 7.16)
Weight^a^ (kg)	Cases Controls	78.0 ± 14.2 70.0 ± 12.3	7.91 (4.63, 11.2)	82.1 ± 14.6 73.1 ± 13.4	9.02 (5.54, 12.5)	86.0 ± 15.2 76.0 ± 14.1	10.0 (6.38, 13.6)	84.5 ± 14.2 77.0 ± 16.7	7.50 (1.88, 13.1)	84.5 ± 16.5 76.8 ± 15.0	7.63 (1.61, 13.7)
Parity^b^	Cases Controls	2.88 ± 1.56 2.43 ± 1.54	0.46 (− 0.08, 0.99)	2.97 ± 1.50 2.55 ± 1.46	0.42 (− 0.09, 0.93)	2.97 ± 1.50 2.60 ± 1.45	0.37 (− 0.14, 0.87)	2.89 ± 1.28 2.65 ± 1.75	0.24 (− 0.47, 0.95)	2.66 ± 1.32 2.76 ± 1.39	− 0.11 (− 0.80, 0.58)
Breastfeeding^c^ (months)	Cases Controls	NA NA	NA	12.7 ± 11.0 12.0 ± 11.1	0.64 (− 3.40, 4.67)	13.5 ± 12.1 14.0 ± 11.7	− 0.49 (− 4.77, 3.79)	13.3 ± 13.3 14.1 ± 14.7	− 0.86 (− 7.88, 6.16)	11.3 ± 8.87 18.0 ± 14.8	− 6.75 (− 13.2, − 0.27)
Body Mass Index^d^ (kg/m^2^)	Cases Controls	27.3 ± 3.91 24.2 ± 3.34	3.15 (2.25, 4.04)	29.0 ± 4.27 25.4 ± 3.97	3.61 (2.59, 4.63)	30.6 ± 4.79 26.5 ± 4.15	4.05 (2.94, 5.15)	30.8 ± 4.78 27.3 ± 4.91	3.46 (1.70, 5.21)	30.5 ± 5.82 27.1 ± 4.48	3.33 (1.36, 5.30)
Total lipids (g/L)	Cases Controls	8.05 ± 1.84 7.15 ± 1.39	0.90 (0.51, 1.30)	8.30 ± 1.82 7.58 ± 2.05	0.72 (0.23, 1.20)	7.53 ± 1.33 7.10 ± 1.29	0.43 (0.11, 0.76)	7.33 ± 1.49 7.25 ± 1.31	0.08 (− 0.42, 0.59)	6.35 ± 1.47 6.59 ± 1.21	− 0.24 (− 0.75, 0.27)
Education level (1–5)^e^	Cases Controls	1.72 ± 1.08 2.02 ± 1.26	− 0.30 (− 0.60, 0.01)	1.98 ± 1.19 2.19 ± 1.39	− 0.20 (− 0.53, 0.12)	1.90 ± 1.22 2.24 ± 1.32	− 0.31 (− 0.73, 0.09)	2.05 ± 1.25 2.34 ± 1.51	− 0.29 (− 0.69, 0.11)	1.71 ± 0.85 2.04 ± 1.15	− 0.34 (− 0.72, 0.05)

		Active, n (%)	Inactive, n (%)	Active, n (%)	Inactive, n (%)	Active, n (%)	Inactive, n (%)	Active, n (%)	Inactive, n (%)	Active, n (%)	Inactive, n (%)
Physical activity	Cases Controls	94 (81) 111 (80)	22 (19) 28 (20)	64 (56) 98 (71)	51 (44) 39 (29)	73 (63) 95 (68)	43 (37) 44 (32)	42 (74) 53 (84)	15 (26) 10 (13)	31 (62) 53 (91)	17 (38) 5 (9)

n = 255 with 116 cases at T1; n = 252 with 115 cases at T2; n = 255 with 116 cases at T3; n = 120 with 57 cases at T4; n = 108 with 50 cases at T5; NA: Not available.

^a,^^d^n = 1 for missing values.

^b,^^c^Only in women: T1-T3: n = 135, 60 cases; T4: n = 76, 36 cases; T5: n = 63, 29 cases.

^e^Education levels 1: 7–10 years primary/secondary school, 2: Technical school, middle school or vocational school, 1–2 years senior high school, 3: High school diploma (3–4 years), 4: College or university (< 4 years), 5: College/university (4 years or more).

### PFAS concentrations in cases versus controls

**Fig. 2:**
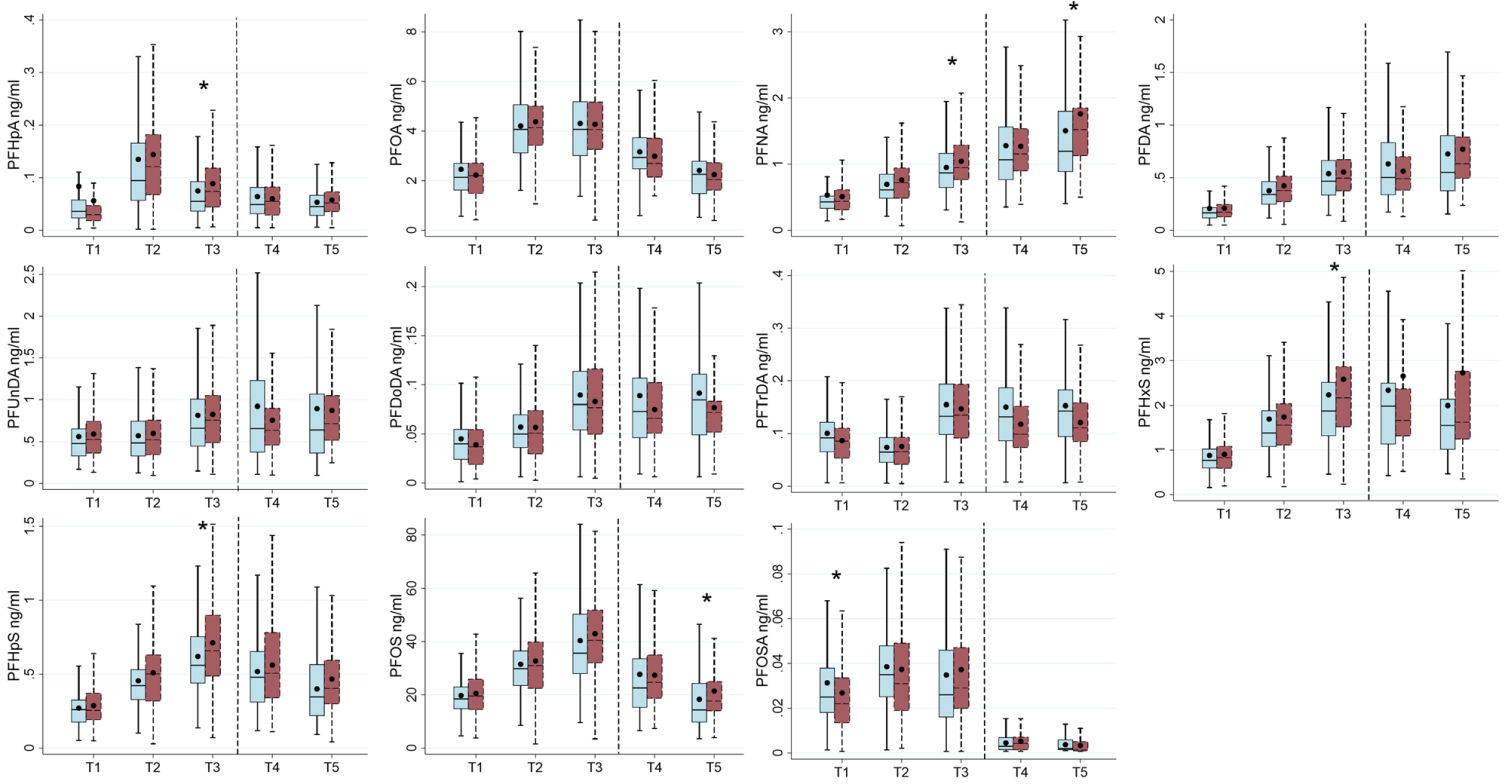
PFAS concentrations in type 2 diabetes mellitus cases (red dashed boxes and whiskers) and controls (blue solid boxes and whiskers) at five time-points (T) in the Tromsø Study (1986–2016). T1-1986/87 (n = 255, 116 cases); T2-1994/95 (n = 252, 115 cases); T3-2001 (n = 255, 116 cases); T4-2007/08 (n = 120, 57 cases); T5-2015/16 (n = 108, 50 cases). Boxes show the 25th–75th percentiles, horizontal lines within the boxes show the median, whiskers indicate 1.5 times the length of the interquartile range above and below the 75th and 25th percentiles, respectively, • indicates the mean, * indicates *p* < 0.05 (Mann Whitney U test). Pre- and post- diagnostic time-points are divided by the vertical stippled lines.

Eleven PFAS were detected above MDL in over 70% of the blood samples at all time-points (Supplemental Table S1). There were positive correlations between all the PFAS compounds at each time-point and these ranged from 0.09 to 0.93. PFOS and PFHpS correlated most at all time-points, whereas the lowest correlation was observed between PFHpA and PFOSA at T5 (Supplemental Table S2). The highest concentrations at all time-points, in descending order, were found for PFOS followed by PFOA, PFHxS, PFNA and PFUNDA, whereas the lowest concentrations were found for PFHpS, PFDA, PFTrDA, PFDoDA, PFHpA and PFOSA in varying order according to time-point (Fig. 2 and Supplemental Table S3). At the pre-diagnostic time-points T1 and T2, concentrations were similar between cases and controls, except for PFOSA, which had significantly lower median concentrations in cases compared to controls at T1 (0.02 ng/ml vs. 0.03 ng/ml, respectively). At T3, concentrations of PFHpA, PFNA, PFHxS and PFHpS were significantly higher in cases than in controls (0.07 vs. 0.06, 0.95 vs. 0.87, 2.17 vs. 1.88, and 0.66 vs. 0.56 ng/ml, respectively). At the post-diagnostic time-points T4 and T5, concentrations were similar between cases and controls, with the exception of PFNA, PFOS and ∑PFSA at T5, which were higher in cases compared to controls (1.52 vs. 1.19 ng/ml, 17.7 vs. 14.3, and 20.3 vs. 16.3, respectively).

### Associations between PFAS and T2DM

**Fig. 3:**
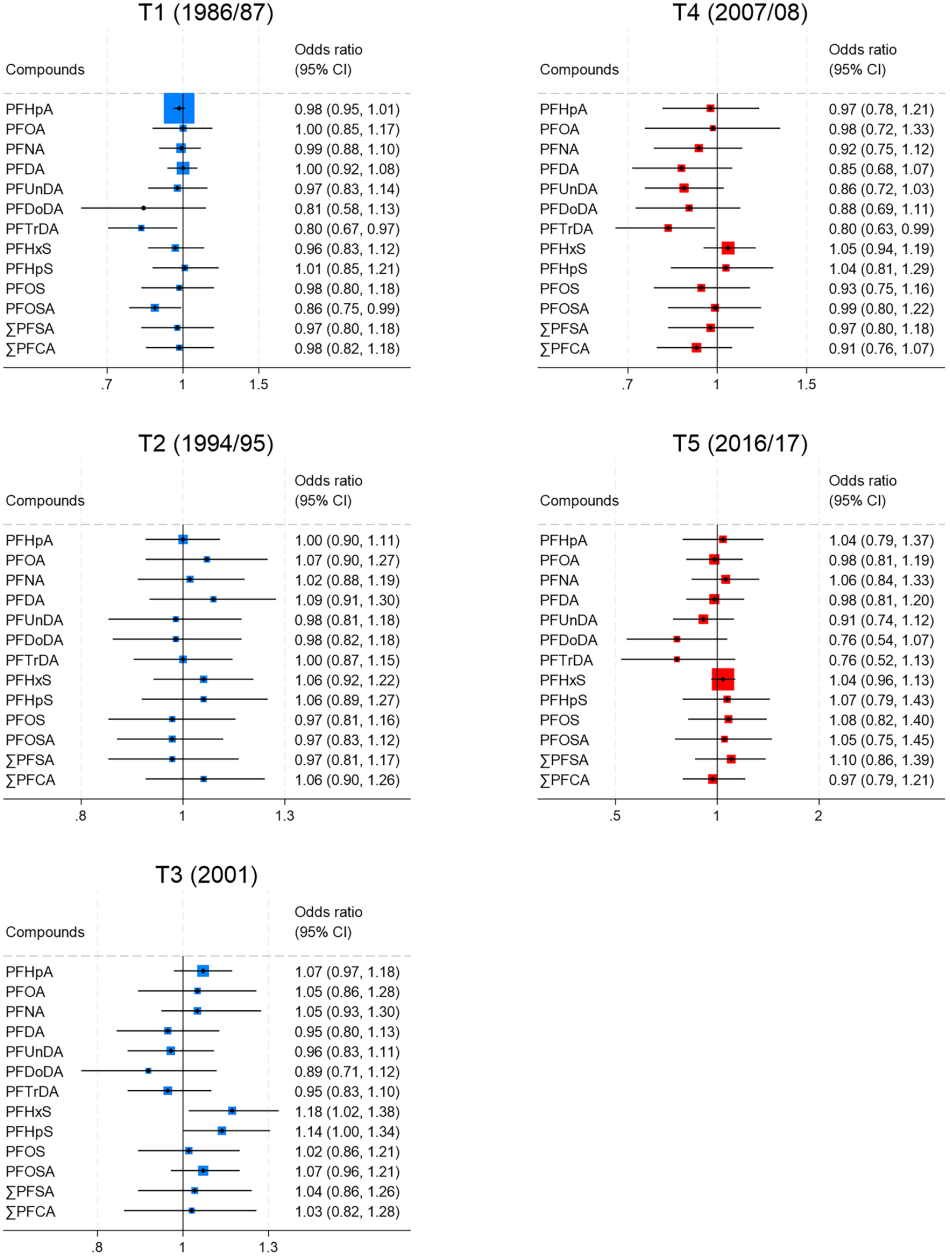
Odds ratios and confidence intervals (95% CIs) for the association per one quartile increase in concentrations of perfluoroalkyl substances and type 2 diabetes mellitus at time-point 1 to 5 (T1–T5) in The Tromsø Study (1986–2017). Adjustments were made for sex, age, BMI, weight change, parity, breastfeeding, physical activity, and education level. Colored squares around the mean represent the weight of the estimate. The weights are based on the inverse variance of the estimates and larger boxes indicate more precise estimates.

After adjusting for the confounders sex, age, BMI, weight change, parity, breastfeeding, physical activity, and education level, PFHxS and PFHpS were positively associated with T2DM at T3 (OR 1.18 95% CI 1.02–1.38 for PFHxS, and OR 1.14 95% CI 1.00, 1.32), whereas, concentrations of PFTrDA were inversely associated with T2DM at T1 (OR 0.81 95% CI 0.67–0.97) and T4 (OR 0.80 95% CI 0.63–0.99) (Fig. 3 and Supplemental Table S4). There was no significant association with T2DM for the other PFAS concentrations, neither between ∑PFCA or ∑PFSA and T2DM at any time-point. These results were similar when stratifying the analyses by sex and age group (results not reported).

### Longitudinal changes in concentrations of PFAS in cases and controls

**Fig. 4:**
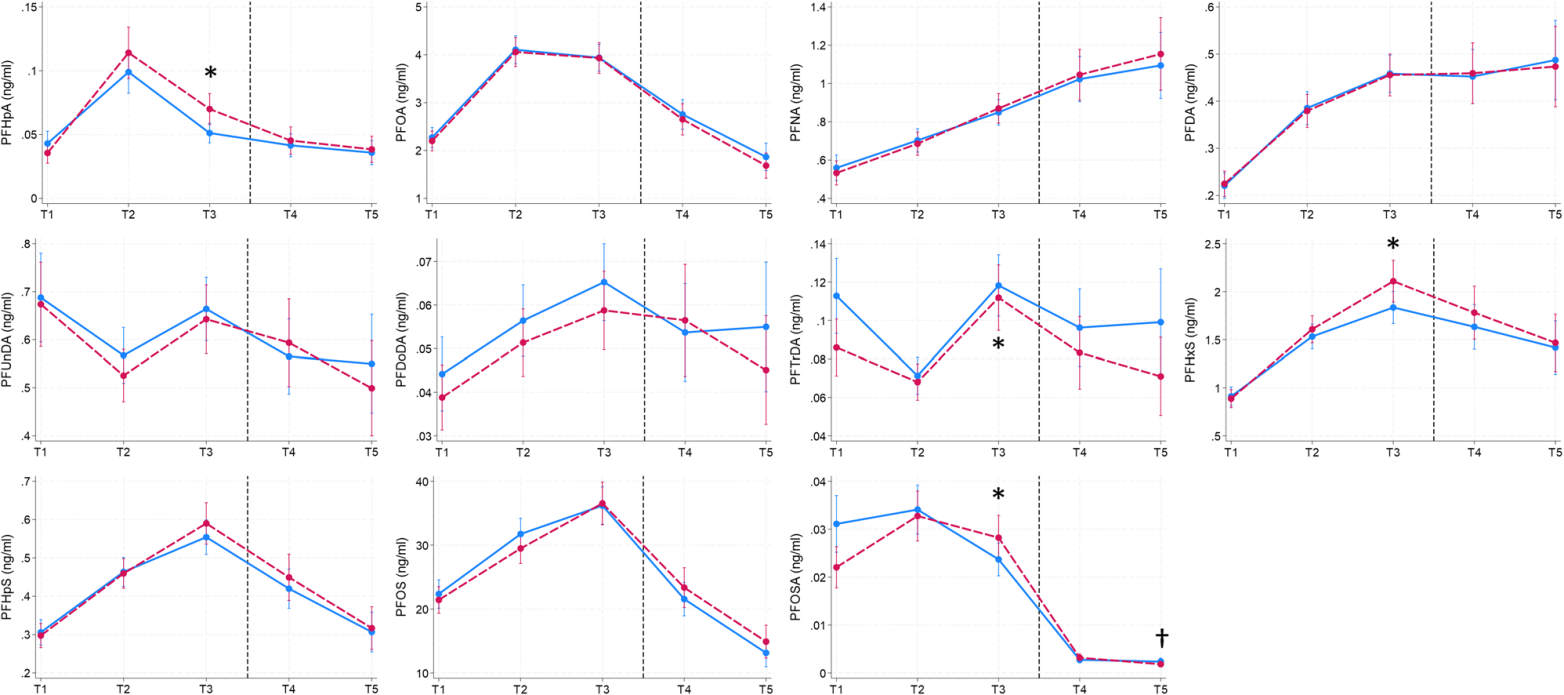
Estimated concentrations from linear mixed models of perfluoroalkyl substances in type 2 diabetes mellitus cases (red dashed lines) and controls (blue lines) from time-point (T) 1 to T3 and from T3 to T5 from models adjusting for the covariates sex, age, BMI, weight change, parity, breastfeeding, physical activity, and education level in the Tromsø Study (1986–2016) (n = 990). T1-1986/87; T2-1994/95; T3-2001; T4-2007/08; T5-2015/16. The vertical stippled lines separate the pre- and post- diagnostic time-points. *Denotes significant increase from T1 to T3 in future cases, †Denotes significant decrease form T3 to T5 in cases.

The unadjusted longitudinal changes in PFAS concentrations over the study period were largely similar for both cases and controls (Supplemental Table S5). Concentrations of PFUnNDA, PFDoDA, PFTrDA, PFHxS, PFHpS, PFOS and PFOSA increased from T1 to T3 (PFUnDA and PFOSA increased and decreased from T1 to T2, and T2 to T3, respectively) proceeded by a decrease from T3 to T4 and T5. For PFHpA and PFOA, concentrations increased from T1 to T3 but peaked at T2 before declining from T2 to T3, T4, and T5. Meanwhile, concentrations of PFNA and PFDA continued to increase throughout the study period. After adjusting for sex, age, BMI, weight change, parity, breastfeeding, physical activity, and education level, cases exhibited larger increases in PFHpA, PFTrDA, PFHxS and PFOSA from T1 to T3, and a more marked decrease in PFOSA from T3 to T5 compared to controls (Fig. 4). The results remained consistent when stratifying the analyses by sex and age groups (results not reported).

## Discussion

To the best of our knowledge, this is the first study to investigate the associations between PFAS and T2DM over 30 years, including multiple repeated pre- and post- diagnostic PFAS measurements within the same individuals. This design offers new insight into the temporal relationship between PFAS and T2DM, which have not been explored in previously published studies. Our study reports positive and negative associations between certain PFAS and T2DM over the study period, demonstrating differences in associations according to year of sample collection and the specific PFAS compounds. For instance, concentrations of PFHxS and PFHpS were positively associated with the development of T2DM at T3, while concentrations of PFTrDA were inversely associated with the development of T2DM at T1 and prevalent T2DM at T4. The overall changes in PFAS concentrations from 1986 to 2016 were mostly similar between cases and controls, although cases appeared to have a more rapid increase in pre-diagnostic concentrations of PFHpA, PFTrDA, PFHxS, PFHpS (95% CIs included 0 for PFHpS) and PFOSA, and a more rapid decrease in post-diagnostic concentrations of PFOSA compared to controls. This may indicate a difference in the rate of accumulation and/or elimination of PFAS in cases compared to controls.

In our study analyses using the pre-diagnostic time-points showed that many PFAS compounds were not associated with the development of T2DM which both support and contradict findings from other prospective studies^12,18–21^. However, we found concentrations of PFHxS and PFHpS at T3 (2001) to be positively associated with the development of T2DM, which is consistent with results reported by Park et al.^20^. They observed a positive association between PFHxS and the risk of developing T2DM when comparing the highest to the lowest tertiles (HR 1.58 95% CI 1.13, 2.21) in women from the US. In their study, 1237 participants donated blood in 1999–2000 and were followed up until 2017, with 102 developing T2DM; they did not measure PFHpS. PFHxS has also been linked to the development of T2DM in American youths (n = 445), where PFHxS at baseline were found to be positively associated with dysregulated glucose metabolism during puberty and post puberty in females^22^. Further, our findings for PFHxS in this study are inconsistent with our previous study, which included pre- and a post-diagnostic concentration of PFAS (sampled in 2001/02 and 2005/06, respectively) in a case–control study (46 cases and 85 controls) from the Norwegian Women and Cancer Study^12^. In that study we observed no significant associations between any PFAS and the development of T2DM. Conversely, Park et al. also reported positive associations between PFOA and PFOS and the development of T2DM,which contrasts with our results, and in a follow up study of children in the Faroe Islands born in 1986–1987, prenatal exposure to PFOS were associated with decreased insulin sensitivity increased beta-cell function at later ages^23^. Similarly, positive associations between PFOS and PFOA concentrations and T2DM were reported in a prospective nested case–control study involving 1586 US women during the period 1995–2000, in which 793 participants developed T2DM (with a follow-up 6.7 ± 3.7 years)^21^. In comparison, although the PFAS concentrations at T3 in our study sample were comparable to the concentrations in US women, the sample size in the studies varies and may explain some of the differences in results.

In the current study, cross-sectional analyses using the post-diagnostic time-points showed that none of the PFAS compounds were positively associated with prevalent T2DM, which aligns with the findings from our previous study in women^12^ and from a cross-sectional study with participants form the C8 Health Project exposed to PFOA through drinking water, where PFOA was not associated with T2DM or fasting glucose levels in prevalent cases (n = 1055)^24^.However, this stands in contrast to other cross-sectional studies that have demonstrated positive associations between PFAS compounds and prevalent T2DM^25–27^. For instance, a study from Korea found that prevalent T2DM cases (n = 44) had higher concentrations of PFHxS and PFOSA compared to controls (n = 742)^27^, Still, these studies varies in the number of participants, sampling year, and time since diagnosis and this may explain varying results and conclusions.

We observed inverse associations between some PFAS compounds and T2DM, where PFTrDA was significantly inversely associated with developing T2DM (at T1, 15–21 years prior to diagnosis) and with prevalent T2DM (at T4, 1–6 years after diagnosis). However, the concentrations of PFTrDA were low in both cases and controls at all time-points and the CIs for the ORs were wide. Overall, our results are in line with several studies that also have reported both positive and negative associations between different PFAS and T2DMwithin the same study^18,19,24,28,29^. For instance, a study from China including 252 cases and 252 controls, reported positive associations between PFHxS and prevalent T2DM, and at the same time inverse associations between several other PFAS and prevalent T2DM^25^. Further, PFAS were mostly inversely associated with prevalent T2DM in another cross-sectional study from China (152 cases and 152 controls)^29^. In the Korean study sample there were no association between PFTrDA concentrations and T2DM, although certain PFAS, including PFTrDA, were negatively associated with BMI^27^. The different directions in associations between different PFAS and T2DM indicate that they may be related to different risk mechanisms for T2DM and complicate the use of summed PFAS variables or investigating the cumulative effect of PFAS on T2DM.

In the present study, there are inconsistencies regarding which PFAS compounds are associated with T2DM when assessed at different time points. Although pre-diagnostic concentrations of PFHxS and PFHpS were positively associated with the development of T2DM, this was only significant at T3. The post-diagnostic concentrations of PFHxS and PFHpS were also positively associated with being diagnosed with T2DM but not significantly. However, our sample size at T4 and T5 were much lower compared to at T1-T3, hence the associations at T4 and T5 have lower statistical power. Our study did not find an association between PFOS and T2DM, unlike many other studies, even though PFOS concentrations were substantially higher than those of PFHxS and PFHpS. One proposed hypothesis is the existence of inverted U-shaped dose–response relationships between PFAS and T2DM, where even lower concentrations might have adverse effects, as suggested by Dian et al. (2021) for PFHxS and PFHpA. Our data did not exhibit any such patterns at any of the time-points, however our sample size is too small to statistically conclude on this.

In the present study, the temporal change in pre- and post-diagnostic concentrations of certain PFAS were associated with T2DM status, with cases generally exhibiting a more rapid increase in pre-diagnostic concentrations of PFHpA, PFTrDA, PFHxS, PFHpS (95% CIs included 0 for PFHpS), and PFOSA from T1 to T3, and a more rapid decrease in post-diagnostic concentrations of PFOSA from T3 to T5 compared to controls. Concurrently, the temporal trends of PFAS indicated that the concentrations of PFHxS and PFHpS reached their peak in our study sample at T3, a pattern that has also been reported in another study^10^. The rise in PFAS exposure during the study period was observed for both cases and controls. The greater pre-diagnostic increase in cases for some PFAS, could mean that cases have accumulated more of these compounds through their diet compared to controls at the same time-period, or that increased PFAS concentrations were linked to other factors associated with the development of T2DM, for example impaired kidney functions with decrease in glomerulus filtration rate (GFR). Impaired kidney function and a decrease in GFR is associated with diabetes type 2^30^ and may cause a decreased secretion of PFAS through the urine. Accordingly, studies report inverse association between certain PFAS and GFR rate^31,32^. This could potentially explain the positive associations observed for PFHxS and PFHpS with T2DM at T3, however, we did not have information on GFR or development of kidney disease and as such could not adjust for kidney function.

Overall, our study results agree with reports by other studies, observing inverse and positive associations depending on compound, and show that differences in study designs and in the timing of blood collection with respect to T2DM diagnosis may affect the results. Unlike our study, which included three pre-diagnostic and two post-diagnostic measurements, other studies have included a single pre-diagnostic or post-diagnostic measurement. Our study spanned a 30-year period, encompassing significant shifts in historical PFAS exposures, with most PFAS concentrations rising from T1 to T3 due to increased exposure of the general population to PFAS in this period. This means that PFAS measurement at one time-point will not necessarily reflect long-term PFAS exposure, as shown in our data, where a cross-sectional study only including PFAS measurements from T1, would have yielded different conclusions compared to T3 or T4 as to the relationship between some PFAS and T2DM. Probably contributing to the discrepancies observed in associations across many studies based on single PFAS measurements, are that they differ in the year of sampling, time to T2DM diagnosis, and involve different populations with varying exposures. Furthermore, inconsistent findings on the associations between several PFAS and T2DM between studies, may also be a result of the metabolic processes involved in T2DM development that are associated with concentrations of PFAS. Numerous studies report that PFAS are associated with blood lipids^27,33–35^. Therefore, it can be hypothesized that PFAS affect the lipid metabolism which increases the risk of developing T2DM, and/or that changes in lipid metabolism due to T2DM could influence circulating PFAS concentrations. Indeed, in the same study sample as the current study, we have shown that cases and controls have different pre- and post-diagnostic lipid trajectories^36^. As individuals developing T2DM undergo changes in blood lipids both before and after diagnosis (also due to use of lipid lowering medication), it is likely that the associations between PFAS concentrations, BMI, lipids and T2DM are complex to investigate, and studies on the potential mediating effect of lipids in the casual pathway between PFAS and T2DM should be studied in more detail.

In Norway, diet is an important source of PFAS exposure, hence dietary habits during periods of rising exposure will influence individual accumulation of the different PFAS. We have previously reported that fish intake was associated with higher blood concentrations of PFOS, PFNA, PFDA and PFUnDA, whereas meat and salty snacks were associated with higher concentration of PFOA in pregnant women from the same region in Norway as the present study sample^37^. As such, if an unhealthy or healthy diet is higher or lower in different PFAS, the fact that certain dietary habits are associated with T2DM risk means that potential residual or unmeasured confounding may occur. We did not have detailed dietary data from the pre-diagnostic surveys, however, cases and controls reported similar intakes of fish at all time-points (Supplemental Table S6). Further, not all participants attended T4 and/or T5, so the sample sizes at T4 and T5 were smaller compared to T1-T3, which could have affected the precision of the effect estimates for the post-diagnostic concentrations of PFAS and associations with T2DM. In conducting multiple tests in this study, there is a possibility of generating type 1 errors, where results appear significant by chance. According to Bender et al., applying proper correction for multiple testing in exploratory studies is challenging and not necessary^38^, however, our study has both exploratory and confirmatory characteristics, hence we assessed our results with caution and adjusted for multiple comparisons to avoid overinterpretation of our findings, as Bender et al. recommends. The 99.5 CIs for all the models included 1, demonstrating non significance at a 0.005 significance level, and studies with larger sample sizes are warranted. Lastly, the generalization of our findings may be limited to populations like the adult Norwegian population.

The extensive study period and the repeated measurements, spanning up to 21 years before diagnosis and 21 years after in cases, constitute a significant strength of the study. This innovative design has allowed us to observe temporal changes between cases and controls and to assess the relationship between PFAS and T2DM both prospectively and cross-sectionally within the same individuals. Consistent with findings that PFAS are associated with lipids, diabetes- and inflammation markers which represent processes increasing the risk of developing insulin resistance and eventually T2DM^6,33,39–43^, and that impaired kidney function related to disease may effect PFAS concentrations^31,32^, we propose that further investigations into any potential relationship between PFAS and T2DM should include longitudinal studies on processes leading to T2DM and whether PFAS are related to early metabolic alterations. This requires more longitudinal prospective studies with repeated measurements of both PFAS and metabolic markers.

## Conclusions

This is presently the only study available which has studied PFAS and T2DM using multiple repeated measurements of PFAS in the same individuals over a period of 30 years. Our results demonstrate: i) differences in concentrations and temporal changes of some PFAS between T2DM cases and controls and, ii) associations between certain PFAS and T2DM which varied according to time-point. This implies that there may be a temporal element in the relationship between PFAS and T2DM, where observed concentrations, changes and associations are influenced by blood collection year and temporal patterns of PFAS exposure. Our results underscore the challenges in studying PFAS as risk factors for T2DM, and more longitudinal studies with repeated measurements are warranted.

## Supplementary Information

Supplementary Information.

## Data Availability

The data that support the findings of this study are available from The Tromsø Study registry, but restrictions apply to the availability of these data, which were used under license for the current study, and so are not publicly available. However, data are available from the corresponding author upon reasonable request and with the permission of The Tromsø Study. Participation in The Tromsø Study was voluntarily, and a signed informed consent form was obtained from all participants.

## Abbreviations

BMI: Body mass index
CI: Confidence interval
DAG: Directed acyclic graph
MDL: Method detection limit
MRM: Multiple reaction monitoring
PFAS: Perfluoroalkyl substances
PFDA: Perfluorodecanoic acid
PFCA: Perfluoro carboxylic acids
PFDoDA: Perfluorododecanoic adic
PFHpA: Perfluoroheptanoic acid
PFHpS: Perfluoroheptane sulfonic acid
PFHxS: Perfluorohexane sulfonic acid
PFNA: Pefluorononanoic acid
PFOA: Perfluorooctanoic acid
PFOS: Perfluorooctane sulfonic acid, P
FOSA: Perfluorooctane sulfonamide
PFTrDA: Perfluorotridecanoic acid
PFSA: Perfluorosulfonic acids
PFUnDA: Perfluoroundecanoic
SD: Standard deviation
T2DM: Type 2 diabetes mellitus
