# Supplementary material for: Temporal changes in per and polyfluoroalkyl substances and their associations with type 2 diabetes
Source: Sci Rep. 2025 Jul 1;15:22026. doi: 10.1038/s41598-025-05422-1 (PMC12219690; doi:10.1038/s41598-025-05422-1)
Supplement: Supplementary file 1 — Supplementary Information. [file 41598_2025_5422_MOESM1_ESM.docx]

**Supplemental material**

**Temporal changes in per and polyfluoroalkyl substances and their associations with type 2 diabetes**

Vivian Berg^1,2*^, Dolley D. Charles^3^, Sandra Huber^2^, Therese H. Nøst^3,4^, Torkjel M. Sandanger^3,5^, Maria Averina^2^, Ingvar A Bergdahl^6^, Mia M. Nilsen^1^, Tom Wilsgaard^3^, Charlotta Rylander^3^

^1^Department of Medical Biology, Faculty of Health Sciences, UiT - The Arctic University of Norway, Tromsø, Norway

^2^Department of Laboratory Medicine, University Hospital of North-Norway, Tromsø, Norway

^3^Department of Community Medicine, Faculty of Health Sciences, UiT - The Arctic University of Norway, Tromsø, Norway

^4^Department of Public Health and Nursing, HUNT Research Centre, Norwegian University of Science and Technology, NO 7491 Trondheim, Norway

^5^The Climate and Environmental Research Institute NILU, Tromsø, Norway

^6^Department of Public Health and Clinical Medicine, Section of Sustainable Health, Umeå University, Umeå, Sweden.

*Correspondence:

Vivian Berg, Department of Medical Biology, P.O Box 6050 Stakkevollan, Faculty of Health Sciences, UIT-The Arctic University of Norway, NO-9037 Tromsø, Norway.

ORCID ID: 0000-0001-5620-9901

Phone: 00 47 77 64 46 77

Email address: Vivian.berg@uit.no

Table of content

**Table S1.** Detection frequencies of the perfluoroalkyl substances at five time-points (T) in the Tromsø Study (1986-2016)

**Table S2**: Spearman`s rank correlation between the different perfluoro alkyl substances at each time-point (T) in the Tromsø Study (1986-2016)

**Table S3:** Concentrations of perfluoroalkyl substances, presented as means with standard deviations (SD), medians and minimum and maximum concentrations for cases and controls, and the p value for the difference in concentrations between type 2 diabetes mellitus cases and controls at each time-point (T). The Tromsø study (1986-2016)

**Table S4**. Odds ratios (ORs), and 95% confidence intervals (CIs) for the associations between one quartile increase in concentrations of perfluoroalkyl substances and type 2 diabetes mellitus (T2DM) at different time-points (T) in the Tromsø Study (1986-2016)

**Table S5.** Crude and multivariate adjusted regression coefficients, standard errors (SE) and 95% confidence intervals (CIs) from linear mixed effect models studying the longitudinal changes in perfluoroalkyl substances (ng/ml) according to type 2 diabetes mellitus (T2DM status (n=990) from 1986-2016 in the Tromsø Study

**Table S6.** Reported intake of fatty and lean fish presented as means and standard deviations (SD) for cases and controls, and the mean difference (Δ) between type 2 diabetes mellitus cases and controls at each pre- and post-diagnostic time-points (T). The Tromsø study (1986-2016)

**Figure S1.** The causal relationship between perfluoroalkyl substances (PFAS) and type 2 diabetes mellitus (T2DM) and potential confounders, illustrated in a directed acyclic graph (DAG)

**Table S1: Detection frequencies and method detection limit (MDL) of the perfluoroalkyl substances at different time-points (T) in the Tromsø Study (1986-2016)**

|  |  | Pre-diagnostic time-points | | | Post-diagnostic time-points | |
| --- | --- | --- | --- | --- | --- | --- |
| **Compound** | **MDL ng/ml** | **T1 (1986/87)**  **n=290**  **%** | **T2 (1994/95)**  **n=290**  **%** | **T3 (2001)**  **n=290**  **%** | **T4 (2007/08)**  **n=130**  **%** | **T5 (2015/16)**  **n=122**  **%** |
| PFHxA | 0.014 | 80 | 40 | 65 | 97 | 93 |
| PFHpA | 0.008 | 87 | 98 | 95 | 98 | 97 |
| PFOA | 0.053 | 100 | 100 | 100 | 100 | 100 |
| PFNA | 0.008 | 100 | 100 | 100 | 100 | 100 |
| PFDA | 0.009 | 100 | 100 | 100 | 100 | 100 |
| PFUnDA | 0.011 | 100 | 100 | 100 | 100 | 100 |
| PFDoDA | 0.013 | 82 | 99 | 88 | 95 | 97 |
| PFTrDA | 0.017 | 93 | 98 | 93 | 96 | 96 |
| PFTeDA | 0.018 | 2 | 16 | 1 | 53 | 58 |
| PFBS | 0.004 | 1 | 8 | 2 | 53 | 63 |
| PFPS | 0.003 | 40 | 52 | 70 | 74 | 77 |
| Linear PFHxS | 0.007 | 100 | 100 | 100 | 100 | 100 |
| Sum PFHxS | 0.007 | 100 | 100 | 100 | 100 | 100 |
| Linear PFHpS | 0.003 | 100 | 100 | 100 | 100 | 100 |
| Sum PFHpS | 0.003 | 100 | 100 | 100 | 100 | 100 |
| Linear PFOS | 0.016 | 100 | 100 | 100 | 100 | 100 |
| Sum PFOS | 0.018 | 100 | 100 | 100 | 100 | 100 |
| Linear PFNS | 0.004 | 20 | 43 | 47 | 71 | 70 |
| Sum PFNS | 0.004 | 22 | 44 | 51 | 72 | 71 |
| PFDoDS | 0.003 | 0 | 2 | 0 | 53 | 58 |
| Linear PFOSA | 0.002 | 97 | 98 | 97 | 85 | 80 |
| Sum PFOSA | 0.002 | 97 | 99 | 97 | 85 | 81 |
| 4.2 FTS | 0.007 | 0 | 2 | 0 | 53 | 58 |
| 6.2 FTS | 0.008 | 9 | 4 | 16 | 58 | 59 |
| 8.2 FTS | 0.005 | 13 | 70 | 70 | 72 | 69 |
| 10.2 FTS | 0.005 | 5 | 18 | 12 | 55 | 58 |

**Table S2. Spearman`s rank correlation between the different perfluoroalkyl substances at each time-point (T) in the Tromsø Study (1986-2016)**

|  | **Pre-diagnostic time-points** | | | **Post-diagnostic time-points** | |
| --- | --- | --- | --- | --- | --- |
| Compound | **T1 (1986/87)** | **T2 (1994/95)** | **T3 (2001)** | **T4 (2007/08)** | **T5 (2015/16)** |
| **PFHpA** |  |  |  |  |  |
| PFOA | 0.3907 | 0.4349 | 0.4329 | 0.4592 | 0.4824 |
| PFNA | 0.2979 | 0.1645 | 0.2344 | 0.3752 | 0.4243 |
| PFDA | 0.1867 | 0.1194 | 0.1688 | 0.3007 | 0.3447 |
| PFUnDA | 0.0906 | 0.1122 | 0.1479 | 0.2158 | 0.2673 |
| PFDoDA | 0.1819 | 0.1147 | 0.1626 | 0.2714 | 0.1981 |
| PFTrDA | 0.1189 | 0.1575 | 0.1504 | 0.2418 | 0.1748 |
| PFHxS | 0.1855 | 0.1419 | 0.1852 | 0.2547 | 0.3297 |
| PFHpS | 0.179 | 0.1453 | 0.1986 | 0.3447 | 0.3479 |
| PFOS | 0.1553 | 0.1888 | 0.2071 | 0.3646 | 0.3983 |
| PFOSA | 0.2341 | 0.2706 | 0.3724 | 0.2761 | 0.0871 |
| **PFOA** |  |  |  |  |  |
| PFNA | 0.4719 | 0.3813 | 0.4814 | 0.5523 | 0.54 |
| PFDA | 0.3907 | 0.4349 | 0.4329 | 0.4592 | 0.4824 |
| PFUnDA | 0.1304 | 0.1548 | 0.27 | 0.2709 | 0.2714 |
| PFDoDA | 0.2519 | 0.1378 | 0.2207 | 0.3179 | 0.2321 |
| PFTrDA | 0.0947 | 0.1424 | 0.1945 | 0.2572 | 0.2177 |
| PFHxS | 0.4889 | 0.3912 | 0.4442 | 0.5913 | 0.5135 |
| PFHpS | 0.5579 | 0.4514 | 0.5715 | 0.6428 | 0.526 |
| PFOS | 0.5579 | 0.4686 | 0.5445 | 0.6126 | 0.5192 |
| PFOSA | 0.2751 | 0.3017 | 0.4275 | 0.2609 | 0.1693 |
| **PFNA** |  |  |  |  |  |
| PFDA | 0.2979 | 0.1645 | 0.2344 | 0.3752 | 0.4243 |
| PFUDA | 0.6818 | 0.7696 | 0.8338 | 0.8105 | 0.7851 |
| PFDoDA | 0.6194 | 0.6612 | 0.7191 | 0.7526 | 0.5396 |
| PFTrDA | 0.5296 | 0.6567 | 0.6592 | 0.7012 | 0.4733 |
| PFHxS | 0.6585 | 0.6642 | 0.6595 | 0.7271 | 0.6865 |
| PFHpS | 0.6555 | 0.7306 | 0.789 | 0.8086 | 0.7625 |
| PFOS | 0.6611 | 0.7567 | 0.8461 | 0.8874 | 0.889 |
| PFOSA | 0.1425 | 0.1657 | 0.4777 | 0.3731 | 0.1902 |
| **PFDA** |  |  |  |  |  |
| PFUnDA | 0.0906 | 0.1122 | 0.1479 | 0.2158 | 0.2673 |
| PFDoDA | 0.1819 | 0.1147 | 0.1626 | 0.2714 | 0.1981 |
| PFTrDA | 0.1189 | 0.1575 | 0.1504 | 0.2418 | 0.1748 |
| PFHxS | 0.1855 | 0.1419 | 0.1852 | 0.2547 | 0.3297 |
| PFHpS | 0.1790 | 0.1453 | 0.1986 | 0.3447 | 0.3479 |
| PFOS | 0.1553 | 0.1888 | 0.2071 | 0.3646 | 0.3983 |
| PFOSA | 0.2341 | 0.2706 | 0.3724 | 0.2761 | 0.0871 |
| **PFUnDA** |  |  |  |  |  |
| PFDoDA | 0.7157 | 0.8798 | 0.8615 | 0.9021 | 0.8066 |
| PFTrDA | 0.8176 | 0.8898 | 0.8227 | 0.8375 | 0.701 |
| PFHxS | 0.5525 | 0.5593 | 0.5606 | 0.608 | 0.5715 |
| PFHpS | 0.5596 | 0.6228 | 0.6353 | 0.5953 | 0.5833 |
| PFOS | 0.6354 | 0.7197 | 0.7757 | 0.7595 | 0.7648 |
| PFOSA | 0.0932 | 0.255 | 0.4821 | 0.2892 | 0.2407 |
| **PFDoDA** |  |  |  |  |  |
| PFTrDA | 0.7191 | 0.8975 | 0.868 | 0.8603 | 0.803 |
| PFHxS | 0.439 | 0.4778 | 0.4608 | 0.6187 | 0.4242 |
| PFHpS | 0.4349 | 0.5014 | 0.5355 | 0.555 | 0.4001 |
| PFOS | 0.4787 | 0.5894 | 0.6492 | 0.6827 | 0.5387 |
| PFOSA | 0.1423 | 0.2515 | 0.4029 | 0.2905 | 0.3199 |
| **PFTrDA** |  |  |  |  |  |
| PFHxS | 0.4485 | 0.5036 | 0.4842 | 0.5014 | 0.3741 |
| PFHpS | 0.4692 | 0.5241 | 0.505 | 0.4978 | 0.343 |
| PFOS | 0.5108 | 0.6025 | 0.6008 | 0.6369 | 0.4662 |
| PFOSA | 0.1292 | 0.2309 | 0.3995 | 0.2033 | 0.3178 |
| **PFHxS** |  |  |  |  |  |
| PFHpS | 0.8971 | 0.8596 | 0.7949 | 0.8302 | 0.8383 |
| PFOS | 0.8142 | 0.7846 | 0.7204 | 0.7771 | 0.7731 |
| PFOSA | 0.1142 | 0.1998 | 0.3900 | 0.3253 | 0.2959 |
| **PFHpS** |  |  |  |  |  |
| PFOS | 0.9331 | 0.9304 | 0.9198 | 0.9244 | 0.9074 |
| PFOSA | 0.1969 | 0.2847 | 0.4871 | 0.4308 | 0.2959 |
| **PFOS** |  |  |  |  |  |
| PFOSA | 0.2324 | 0.3052 | 0.5350 | 0.4145 | 0.3072 |

T1: n=255, 116 cases; T2: n=252, 115 cases; T3: n=255, 116 cases; T4: n=120, 57cases; T5: n=108, 50 cases

**Table S3. Concentrations of perfluoroalkyl substances, presented as mean with standard deviations (SD), medians and minimum and maximum concentrations for cases and controls, and the p value for the difference in concentrations between type 2 diabetes mellitus cases and controls at each time-point (T). The Tromsø study (1986-2016)**

|  |  | **Pre-diagnostic time-points** | | | | | | | | | **Post-diagnostic time-points** | | | | | | |
| --- | --- | --- | --- | --- | --- | --- | --- | --- | --- | --- | --- | --- | --- | --- | --- | --- | --- |
|  |  | **T1 (1986/87)** | | | **T2 (1994/95)** | | |  | **T3 (2001)** | | **T4 (2006/7)** | | | **T5 (2015/16)** | | | |
| **Perfluoroalkyl substance (ng/ml)** |  | **Mean (SD)** | **Median**  **(min, max)** | **p^a^** | **Mean (SD)** | **Median**  **(min, max)** | **p^a^** | **Mean**  **(SD)** | **Median**  **(min, max)** | **p^a^** | **Mean**  **(SD)** | **Median (Min,max)** | **p^a^** | **Mean**  **(SD)** | **Median**  **(min, max)** | **p^a^** |  |
| **PFHpA** | Cases  Controls | 0.06  (0.11)  0.08  (0.25) | 0.03  (0.004, 0.87)  0.04  (0.003, 2.16) | 0.18 | 0.14  (0.12)  0.14  (0.13) | 0.12  (0.002, 0.78)  0.09  (0.002, 0.75) | 0.14 | 0.09  (0.06)  0.08  (0.07) | 0.07  (0.006, 0.38)  0.06  (0.005, 0.54) | **0.01** | 0.06  (0.04)  0.06  (0.05) | 0.06  (0.005, 0.20)  0.05  (0.005, 0.26) | 0.88 | 0.06  (0.03)  0.05  (0.04) | 0.05  (0.005, 0.16)  0.05  (0.006, 0.21) | 0.19 |  |
| **PFOA** | Cases  Controls | 2.23  (0.95)  2.46  (2.08) | 2.20  (0.42, 6.46)  2.14  (0.57, 19.3) | 0.94 | 4.38  (1.98)  4.21  (1.35) | 4.14  (0.41, 14.0)  4.07  (1.61, 7.87) | 0.97 | 4.27  (1.60)  4.31  (1.70) | 4.07  (0.32, 10.4)  4.07  (1.37, 11.6) | 0.76 | 3.00  (1.09)  3.16  (1.23) | 2.70  (1.40, 5.86)  2.94  (0.59, 8.66) | 0.28 | 2.25  (1.09)  2.41  (2.20) | 2.04  (0.39, 6.38)  2.25  (0.52, 17.5) | 0.93 |  |
| **PFNA** | Cases  Controls | 0.51  (0.32)  0.53  (0.68) | 0.44  (0.16,2.60)  0.43  (0.14, 7.86) | 0.45 | 0.76  (0.42)  0.70  (0.36) | 0.72  (0.07, 3.26)  0.61  (0.21, 3.00) | 0.14 | 1.04  (0.46)  0.95  (0.43) | 0.95  (0.13, 2.60)  0.87  (0.31, 2.63) | **0.04** | 1.27  (0.54)  1.28  (0.71) | 1.16  (0.39, 2.91)  1.07  (0.35, 3.90) | 0.46 | 1.76  (0.91)  1.50  (0.97) | 1.52  (0.50, 4.68)  1.19  (0.40, 4.69) | **0.03** |  |
| **PFDA** | Cases  Controls | 0.21  (0.14)  0.21  (0.31) | 0.17  (0.05, 1.13)  0.17  (0.05, 3.72) | 0.14 | 0.42  (0.24)  0.38  (0.17) | 0.38  (0.06, 1.37)  0.34  (0.12, 1.09) | 0.14 | 0.55  (0.26)  0.54  (0.31) | 0.50  (0.09, 1.49)  0.47  (0.14, 1.97) | 0.22 | 0.56  (0.27)  0.63  (0.42) | 0.49  (0.13, 1.44)  0.50  (0.17, 2.32) | 0.85 | 0.77  (0.46)  0.73  (0.55) | 0.63  (0.24, 2.59)  0.55  (0.16, 2.72) | 0.14 |  |
| **PFUnDA** | Cases  Controls | 0.59  (0.31)  0.56  (0.32) | 0.53  (0.14, 1.60)  0.48  (0.17, 1.93) | 0.27 | 0.60  (0.37)  0.57  (0.35) | 0.52  (0.10, 1.92)  0.49  (0.13, 2.12) | 0.58 | 0.83  (0.47)  0.82  (0.55) | 0.76  (0.11, 2.07)  0.66  (0.15, 3.22) | 0.49 | 0.76  (0.47)  0.92  (0.85) | 0.64  (0.10, 2.58)  0.66  (0.11, 5.35) | 0.84 | 0.87  (0.56)  0.90  (0.78) | 0.72  (0.25, 3.06)  0.64  (0.10, 3.61) | 0.41 |  |
| **PFDoDA** | Cases  Controls | 0.04  (0.03)  0.05  (0.05) | 0.04  (0.004, 0.15)  0.04  (0.001, 0.56) | 0.32 | 0.06  (0.03)  0.06  (0.03) | 0.05  (0.003, 0.19)  0.05  (0.006, 0.21) | 0.73 | 0.08  (0.05)  0.09  (0.06) | 0.08  (0.005, 0.22)  0.08  (0.006, 0.31) | 0.60 | 0.08  (0.05)  0.09  (0.07) | 0.07  (0.006, 0.24)  0.07  (0.009, 0.37) | 0.49 | 0.08  (0.04)  0.09  (0.07) | 0.07  (0.01, 0.20)  0.08  (0.006, 0.31) | 0.30 |  |
| **PFTrDA** | Cases  Controls | 0-09  (0.05)  0.10  (0.05) | 0.09  (0.006, 0.25)  0.09  (0.006, 0.34) | 0.12 | 0.08  (0.04)  0.07  (0.04) | 0.07  (0.005, 0.22)  0.07  (0.006, 0.30) | 0.90 | 0.15  (0.08)  0.15  (0.10) | 0.14  (0.006, 0.43)  0.13  (0.008, 0.50) | 0.85 | 0.12  (0.07)  0.15  (0.11) | 0.10  (0.008, 0.29)  0.13  (0.008, 0.53) | 0.10 | 0.12  (0.06)  0.15  (0.10) | 0.11  (0.008, 0.26)  0.14  (0.006, 0.52) | 0.13 |  |
| **PFHxS** | Cases  Controls | 0.91  (0.45)  0.88  (0.55) | 0.83  (0.20, 2.76)  0.77  (0.16, 4.96) | 0.33 | 1.74  (0.93)  1.70  (1.64) | 1.56  (0.18, 6.17)  1.38  (0.40, 17.7) | 0.13 | 2.58  (1.99)  2.23  (1.82) | 2.17  (0.24, 17.2)  1.88  (0.46, 18.3) | **0.02** | 2.65  (4.66)  2.33  (2.29) | 1.67  (0.52, 35.9)  1.98  (0.43, 15.9) | 0.89 | 2.73  (3.85)  2.00  (1.91) | 1.63  (0.35, 26.3)  1.55  (0.47, 12.3) | 0.14 |  |
| **PFHpS** | Cases  Controls | 0.29  (0.14)  0.27  (0.13) | 0.26  (0.05, 0.10)  0.26  (0.05, 0.80) | 0.26 | 0.51  (0.24)  0.45  (0.20) | 0.50  (0.03, 1.21)  0.42  (0.10, 1.15) | 0.07 | 0.71  (0.35)  0.62  (0.30) | 0.66  (0.07, 2.37)  0.56  (0.14, 1.83) | **0.01** | 0.56  (0.30)  0.52  (0.28) | 0.51  (0.11, 1.71)  0.48  (0.12, 1.52) | 0.44 | 0.47  (0.25)  0.40  (0.23) | 0.40  (0.04, 1.24)  0.35  (0.09, 1.106) | 0.06 |  |
| **PFOS** | Cases  Controls | 20.5  (0.39)  19.7  (7.49) | 19.6  (3.79, 43.3)  18.5  (4.59, 47.3) | 0.52 | 32.7  (14.6)  31.5  (13.0) | 31.0  (1.56, 75.4)  29.8  (8.52, 75.2) | 0.39 | 42.9  (18.2)  40.3  (19.4) | 40.5  (3.46, 108)  35.6  (9.63, 108) | 0.06 | 27.4  (13.2)  27.7  (17.9) | 24.7  (7.34, 68.1)  22.6  (6.51, 90.9) | 0.59 | 21.4  (13.2)  18.3  (13.9) | 17.7  (3.90, 71.6)  14.3  (3.47, 74.4) | **0.02** |  |
| **PFOSA** | Cases  Controls | 0.03  (0.02)  0.03  (0.02) | 0.02  (0.001, 0.15)  0.03  (0.001, 0.14) | **0.03** | 0.04  (0.02)  0.04  (0.02) | 0.03  (0.002, 0.14)  0.04  (0.001, 0.16) | 0.37 | 0.04  (0.03)  0.03  (0.03) | 0.03  (0.001, 0.14)  0.03  (0.001, 0.17) | 0.25 | 0.005  (0.004)  0.004  (0.005) | 0.004  (0.001, 0.02)  0.003  (0.001, 0.03) | 0.19 | 0.003  (0.003)  0.004  (0.003) | 0.001  (0.001, 0.01)  0.002  (0.001, 0.02) | 0.12 |  |
| **∑PFSA** | Cases  Controls | 21.7  (8.88)  20.9  (7.99) | 20.7  (4.04, 46.8)  19.8  (4.83, 49.1) | 0.51 | 35.0  (15.5)  33.7  (13.8) | 33.0  (1.90, 80.7)  32.0  (9.10, 79.2) | 0.40 | 46.2  (19.3)  43.2  (20.4) | 43.9  (3.86, 116)  38.6  (10.4, 115) | 0.06 | 30.6  (16.6)  30.5  (18.9) | 26.7  (10.4, 75.9)  26.4  (7.08, 95.9) | 0.70 | 24.6  (15.1)  20.7  (14.9) | 20.3  (5.16, 84.0)  16.3  (4.34, 78.8) | **0.03** |  |
| **∑PFCA** | Cases  Controls | 3.72  (1.51)  3.99  (3.13) | 3.56  (0.91, 12.5)  3.51  (1.23, 32.8) | 0.87 | 6.43  (2.50)  6.12  (1.86) | 6.11  (0.72, 17.032)  5.96  (2.51, 11.6) | 0.36 | 7.02  (2.29)  6.93  (2.55) | 7.00  (0.85, 13.4)  6.46  (2.29, 16.3) | 0.36 | 5.83  (2.02)  6.30  (2.77) | 5.53  (2.50, 10.3)  5.32  (1.72, 15.1) | 0.60 | 5.91  (2.59)  5.84  (3.90) | 5.42  (1.78, 13.4)  5.00  (1.59, 26.1) | 0.29 |  |

T1: n=255, 116 cases; T2: n=252, 115 cases; T3: n=255, 116 cases; T4: n=120, 57 cases; T5: n=108, 50 cases

^a^p-value from two-sample Wilcoxon rank-sum (Mann-Whitney) test, significant p-values are in bold.

**Table S4. Odds ratios (ORs), and 95 and 99.5% confidence intervals (CIs) for the associations between one quartile increase in concentrations of perfluoroalkyl substances and type 2 diabetes mellitus (T2DM) at different time-points (T) in the Tromsø Study (1986-2016).**

|  |  | **Pre-diagnostic time-points** | | | | **Post-diagnostic time-points** | | |
| --- | --- | --- | --- | --- | --- | --- | --- | --- |
|  |  | **T1** | **T2** | **T3** | **T4** | | **T5** |  |
| **Compound pg/ml** |  | **OR (95% CI)** | **OR (95% CI)** | **OR (95% CI)** | **OR (95% CI)** | | **OR (95% CI)** |  |
| PFHpA | Model 1 | 0.98 (0.95, 1.01) | 1.02 (0.93, 1.12) | 1.06 (0.98, 1.15) | 0.93 (0.79, 1.10) | | 1.06 (0.86, 1.31) |  |
|  | Model 2 | 0.98 (0.93, 1.03) | 1.00 (0.90, 1.11) | 1.07 (0.97, 1.18) | 0.97 (0.78, 1.21) | | 1.04 (0.79, 1.37) |  |
|  | Model 2* | 0.98 (0.93, 1.03) | 1.00 (0.87, 1.16) | 1.07 (0.93, 1.23) | 0.97 (0.71, 1.33) | | 1.04 (0.70, 1.54) |  |
| PFOA | Model 1 | 0.95 (0.85, 1.05) | 1.05 (0.90, 1.22) | 0.96 (0.81, 1.13) | 0.89 (0.71, 1.11) | | 0.94 (0.80, 1.12) |  |
|  | Model 2 | 1.00 (0.85, 1.17) | 1.07 (0.90, 1.27) | 1.05 (0.86, 1.28) | 0.98 (0.72, 1.33) | | 0.98 (0.81, 1.19) |  |
|  | Model 2* | 1.00 (0.85, 1.17) | 1.07 (0.84, 1.36) | 1.05 (0.79, 1.40) | 0.98 (0.63, 1.52) | | 0.98 (0.74, 1.29) |  |
| PFNA | Model 1 | 0.98 (0.92, 1.06) | 1.05 (0.92, 1.21) | 1.09 (0.93, 1.30) | 0.96 (0.82, 1.12) | | 1.08 (0.91, 1.28) |  |
|  | Model 2 | 0.99 (0.88, 1.10) | 1.02 (0.88, 1.19) | 1.05 (0.86, 1.28) | 0.92 (0.75, 1.12) | | 1.06 (0.84, 1.33) |  |
|  | Model 2* | 0.99 (0.88, 1.10) | 1.02 (0.85, 1.27) | 1.05 (0.79, 1.39) | 0.92 (0.69, 1.21) | | 1.06 (0.76, 1.48) |  |
| PFDA | Model 1 | 1.00 (0.95, 1.05) | 1.10 (0.93, 1.28) | 0.98 (0.86, 1.13) | 0.86 (0.72, 1.03) | | 0.98 (0.87, 1.14) |  |
|  | Model 2 | 1.00 (0.92, 1.08) | 1.09 (0.91, 1.30) | 0.95 (0.80, 1.13) | 0.85 (0.68, 1.07) | | 0.98 (0.81, 1.20) |  |
|  | Model 2* | 1.00 (0.92, 1.08) | 1.09 (0.84, 1.41) | 0.95 (0.74, 1.22) | 0.85 (0.62, 1.17) | | 0.98 (0.74, 1.31) |  |
| PFUnDA | Model 1 | 1.00 (0.87, 1.15) | 0.99 (0.84, 1.17) | 0.97 (0.86, 1.09) | 0.88 (0.77, 1.01) | | 0.95 (0.82, 1.10) |  |
|  | Model 2 | 0.97 (0.83, 1.14) | 0.98 (0.81, 1.18) | 0.96 (0.83, 1.11) | 0.86 (0.72, 1.03) | | 0.91 (0.74, 1.12) |  |
|  | Model 2* | 0.97 (0.78, 1.22) | 0.98 (0.75, 1.28) | 0.96 (0.78, 1.18) | 0.86 (0.67, 1.11) | | 0.91 (0.68, 1.23) |  |
| PFDoDA | Model 1 | 0.79 (0.58, 1.06) | 0.93 (0.79, 1.10) | 0.87 (0.72, 1.05) | 0.85 (0.69, 1.03) | | 0.82 (0.65, 1.04) |  |
|  | Model 2 | 0.81 (0.58, 1.13) | 0.98 (0.82, 1.18) | 0.89 (0.71, 1.12) | 0.88 (0.69, 1.11) | | 0.76 (0.54, 1.07) |  |
|  | Model 2* | 0.81 (0.50, 1.30) | 0.98 (0.76, 1.27) | 0.89 (0.64, 1.23 | 0.88 (0.62, 1.23) | | 0.76 (0.47, 1.24) |  |
| PFTrDA | Model 1 | **0.78 (0.65, 0.92)** | 0.98 (0.86, 1.10) | 0.93 (0.82, 1.05) | **0.80 (0.66, 0.97)** | | 0.76 (0.57, 1.01) |  |
|  | Model 2 | **0.80 (0.67, 0.97)** | 1.00 (0.87, 1.15) | 0.95 (0.83, 1.10) | **0.80 (0.63, 0.99)** | | 0.76 (0.52, 1.13) |  |
|  | Model 2* | 0.80 (0.61, 1.05) | 1.00 (0.82, 1.21) | 0.95 (0.78, 1.16) | 0.80 (0.57, 1.13) | | 0.76 (0.44, 1.33) |  |
| PFHxS | Model 1 | 1.00 (0.87, 1.15) | 1.00 (0.93, 1.08) | 1.06 (0.97, 1.16) | 1.00 (0.95, 1.07) | | 1.03 (0.96, 1.09) |  |
|  | Model 2 | 0.96 (0.83, 1.12) | 1.06 (0.92, 1.22) | **1.18 (1.02, 1.38)** | 1.05 (0.94, 1.19) | | 1.04 (0.96, 1.13) |  |
|  | Model 2* | 0.96 (0.77, 1.20) | 1.06 (0.87, 1.29) | 1.18 (0.95, 1.48) | 1.05 (0.90, 1,25) | | 1.04 (0.93, 1.17) |  |
| PFHpS | Model 1 | 1.04 (0.89, 1.21) | 1.12 (0.95, 1.31) | 1.14 (0.99, 1.32) | 1.04 (0.85, 1.28) | | 1.09 (0.88, 1.36) |  |
|  | Model 2 | 1.00 (0.85, 1.19) | 1.06 (0.89, 1.27) | **1.14 (1.00, 1.34)** | 1.02 (0.81, 1.29) | | 1.07 (0.79, 1.43) |  |
|  | Model 2* | 1.00 (0.79, 1.27) | 1.06 (0.83, 1.37) | 1.14 (0.89, 1.45) | 1.02 (0.73, 1.42) | | 1.07 (0.70, 1.64) |  |
| PFOS | Model 1 | 1.02 (0.86, 1.21) | 1.00 (0.86, 1.17) | 1.04 (0.90, 1.20) | 0.93 (0.78, 1.13) | | 1.05 (0.88, 1.26) |  |
|  | Model 2 | 0.98 (0.80, 1.18) | 0.97 (0.81, 1.16) | 1.02 (0.86, 1.21) | 0.93 (0.75, 1.16) | | 1.08 (0.82, 1.40) |  |
|  | Model 2* | 0.98 (0.74, 1.29) | 0.97 (0.75, 1.25) | 1.02 (0.80, 1.30) | 0.93 (0.68, 1.27) | | 1.08 (0.74, 1.58) |  |
| PFOSA | Model 1 | 0.90 (0.80, 1.01) | 0.96 (0.84, 1.10) | 1.02 (0.93, 1.12) | 1.02 (0.87, 1.20) | | 0.85 (0.66, 1.09) |  |
|  | Model 2 | **0.86 (0.75, 0.99)** | 0.97 (0.83, 1.12) | 1.07 (0.96, 1.21) | 0.99 (0.80, 1.22) | | 1.05 (0.75, 1.46) |  |
|  | Model 2* | 0.86 (0.71, 1.05) | 0.97 (0.78, 1.20) | 1.07 (0.91, 1.27) | 0.99 (0.73, 1.33) | | 1.05 (0.65, 1.70) |  |
| ∑PFSA | Model 1 | 1.02 (0.86, 1.20) | 1.00 (0.86, 1.18) | 1.05 (0.90, 1.23) | 0.95 (0.80, 1.13) | | 1.06 (0.90, 1.26) |  |
|  | Model 2 | 0.97 (0.80, 1.18) | 0.97 (0.81, 1.17) | 1.04 (0.86, 1.26) | 0.97 (0.80, 1.18) | | 1.10 (0.86, 1.39) |  |
|  | Model 2* | 0.97 (0.74, 1.28) | 0.97 (0.75, 1.27) | 1.04 (0.79, 1.36) | 0.97 (0.73, 1.28) | | 1.10 (0.78, 1,55) |  |
| ∑PFCA | Model 1 | 0.94 (0.83, 1.07) | 1.05 (0.90, 1.23) | 0.97 (0.81, 1.16) | 0.90 (0.79, 1.03) | | 0.97 (0.83, 1.14) |  |
|  | Model 2 | 0.98 (0.86, 1.11) | 1.06 (0.90, 1.26) | 1.03 (0.82, 1.28) | 0.91 (0.76, 1.07) | | 0.97 (0.79, 1.21) |  |
|  | Model 2* | 0.98 (0.82, 1.18) | 1.06 (0.83, 1.35) | 1.03 (0.75, 1.41) | 0.91 (0.71, 1.16) | | 0.97 (0.72, 1.32) |  |

Model 1: Adjusted for age and sex; Model 2: Adjusted for sex, age, BMI, weight change, parity, breastfeeding, physical activity, and education level; Model 2*: ORs with 99.5% CIs for model 2.

**Table S5. Crude and multivariate adjusted regression coefficients, standard errors (SE) and 95 and 99.5% confidence intervals (CIs) from linear mixed effect models studying the longitudinal changes in perfluoroalkyl substances (ng/ml) according to type 2 diabetes mellitus (T2DM status (n=990) from 1986-2016 in the Tromsø Study.**

|  | **Model 1** | | **Model 2** | | |
| --- | --- | --- | --- | --- | --- |
|  | **β – coefficient**  **(SE)** | **95% CI**  **(p-value for Walds test)** | **β – coefficient**  **(SE)** | **95% CI**  **(p-value for Walds test)** | **Adjusted CIs at 99.5%** |
| **PFHpA**   - T2DM | 0.27 (0.11) | 0.05, 0.48 | 0.31 (0.12) | 0.09, 0.54 | -0.01, 0.64 |
| - Sampling year   T1  T2  T3  T4  T5 | -0.41 (0.08)  0.54 (0.08)  Reference  -0.17 (0.11)  -0.27 (0.11) | -0.57, -0.25  0.38, 0.70  -  -0.39, 0,04  -0.49, -0.05 | -0.11 (0.15)  0.57 (0.11)  -  -0.19 (0.14)  -0.33 (0.17) | -0.40, 0.18  0.35, 0.79  -  -0.46, 0.09  -0.65, -0.002 | -0.52, 0.30  0.26, 0.88  -  -0.58, 0.21  -0.79, 0.14 |
| - Interactions   T2DMxT1  T2DMxT2  T2DMxT3  T2DMxT4  T2DMxT5   - Wald test for interaction term | -0.45 (0.12)  -0.12 (0.12)  Reference  -0.15 (0.16)  -0.08 (0.16) | -0.69, -0.21  -0,36, 0.12  -  -0.46, 0.16  -0.41, 0.24  (0.004) | -0.50 (0.13)  -0.17 (0.13)  -  -0.23 (0.16)  -0.24 (0.18) | -0.76, -0.25  -0.42, 0.08  -  -0.55, 0.10  -0.59, 0.11  (0.003) | -0.87, -0.14  -0.53, 0.18  -  -0.69, 0.24  -0.75, 0.26 |
| - Sex - Age - BMIcat   1  2   - Weight change - Parity - Breastfeeding - Physical activity - Education |  |  | -0.13 (0.15)  0.01 (0.005)  0.002 (0.08)  0.04 (0.11)  -0.004 (0.006)  -0.06 (0.04)  0.004 (0.004)  -0.07 (0.06)  0.04 (0.03) | -0.43, 0.17  0.005, 0.02  -0.15, 0.16  -0.17, 0.25  -0.01, 0.007  -0.14, 0.03  -0.005, 0.01  -0.19, 0.06  -0.02, 0.10 | -0.56, 0.31  0.001, 0.03  -0.22, 0.22  -0.26, 0.34  -0.02, 0.01  -0.18, 0.07  -0.01, 0.02  -0.24, 0.11  -0.04, 0.12 |
| - Constant | -2.92 (0.06) | -3.07, -2.77 | -3.08 (0.18) | -3.44, -2.72 | -3.59, -2.56 |
| **PFOA**   - T2DM | -0.02 (0.05) | -0.12, 0.09 | -0.002 (0.05) | -0.11, 0.10 | -1.15, 0.15 |
| - Sampling year   T1  T2  T3  T4  T5 | -0.63 (0.04)  -0.01 (0.04)  Reference  -0.32 (0.04)  -0.71 (0.05) | -0.71, -0.56  -0.08, 0.07  -  -0.42, -0.22  -0.82, -0.61 | -0.64 (0.07)  -0.02 (0.05)  -  -0.33 (0.07)  -0.67 (0.09) | -0.77, -0.50  -0.12, 0.07  -  -0.46, -0.21  -0.84, -0.50 | -0.83, -0.44  -1.16, 0.12  -  -0.51, -0.15  -0.92, -0.43 |
| - Interactions   T2DMxT1  T2DMxT2  T2DMxT3  T2DMxT4  T2DMxT5   - Wald test for interaction term | -0.02 (0.06)  0.02 (0.06)  Reference  -0.02 (0.07)  0.003 (0.08) | -0.14, 0.09  -0.09, 0.13  -  -0.17, 0.12  -0.15, 0.16  (0.953) | -0.03 (0.06)  -0.01 (0.05)  -  -0.04 (0.08)  -0.10 (0.09) | -0.15, 0.09  -0.12, 0.10  -  -0.18, 0.11  -0.28, 0.08  (0.847) | -0.20, 0.14  -0.15, 0.14  -  -0.25, 0.18  -0.36, 0.16 |
| - Sex - Age - BMIcat   1  2   - Weight change - Parity - Breastfeeding - Physical activity - Education |  |  | 0.01 (0.07)  0.006 (0.002)  -0.14 (0.04)  0.02 (0.05)  -0.001 (0.002)  -0.02 (0.02)  0.001 (0.002)  0.002 (0.03)  0.02 (0.01) | -0.14, 0.15  0.002, 0.01  -0.08, 0.06  -0.07, 0.12  -0.006, 0.003  -0.05, 0.02  -0.003, 0.006  -0.05, 0.06  -0.01, 0.05 | -0.19, 0.22  -0.00, 0.01  -0.11, 0.09  -0.12, 0.16  -0.01, 0.005  -0.07, 0.04  -0.005, 0.001  -0.08, 0.08  -0.02, 0.06 |
| - Constant | 1.39 (0.04) | 1.32, 1.46 | 1.27 (0.09) | 1.10, 1.43 | 1.03, 1.51 |
| **PFNA**   - T2DM | 0.08 (0.06) | -0.04, 0.21 | 0.02 (0.06) | -0.09, 0.14 | -0.14, 0.19 |
| - Sampling year   T1  T2  T3  T4  T5 | -0.68 (0.04)  -0.32 (0.04)  Reference  0.27 (0.05)  0.40 (0.05) | -0.75, -0.61  -0.39, -0.25  1.18, 0.36  0.30, 0.50 | -0.39 (0.07)  -0.21 (0.05)  0.17 (0.06)  0.26 (0.09) | -0.53, -0.25  -0.30, -0.11  0.05, 0.29  0.09, 0.43 | -0.59, -0.18  -0.33, 0.07  -  -0.01, 0.33  0.01, 0.49 |
| - Interactions   T2DMxT1  T2DMxT2  T2DMxT3  T2DMxT4  T2DMxT5   - Wald test for interaction term | -0.06 (0.05)  -0.03 (0.05)  Reference  0.03 (0.07)  0.11 (0.07) | -0.17, 0.04  -0.14, 0.07  -0.11, 0.16  -0.03, 0.26  (0.154) | -0.08 (0.06)  -0.05 (0.05)  -0.002 (0.07)  0.03 (0.09) | -0.19, 0.03  -0.15, 0.05  -0.14, 0.14  -0.14, 0.20  (0.750) | -0.23, 0.09  -0,19, 0.09  -  -0.20, 0.19  -0.22, 0.28 |
| - Sex - Age - BMIcat   1  2   - Weight change - Parity - Breastfeeding - Physical activity - Education |  |  | 0.06 (0.09)  0.02 (0.003)  0.06 (0.04)  0.14 (0.05)  -0.003 (0.002)  -0.05 (0.02)  0.005 (0.002)  0.03 (0.03)  -0.01 (0.01) | -0.10, 0.24  0.01, 0.02  -0.01, 0.13  0.03, 0.24  -0.008, 0.001  -0.09, 0.003  0.000, 0.009  -0.02, 0.08  -0.03, 0.02 | -0.17, 0.31  0.01, 0.23  -0.05, 0.16  -0.01, 0.02  -0.01, 0.003  -0.11, 0.02  -0.002, 0.01  -0.05, 0.11  -0.05, 0.04 |
| - constant | -0.15 (0.04) | -0.23, -0.06 | -0.42 (0.10) | -0.62, -0.23 | -0.70, -0.15 |
| **PFDA**   - T2DM | 0.04 (0.07) | -0.09, 0.17 | -0.01 (0.07) | -0.14, 0.12 | -0.19, 0.18 |
| - Sampling year   T1  T2  T3  T4  T5 | -1.04 (0.04)  -0.32 (0.04)  Reference  0.10 (0.05)  0.23 (0.05) | -1.11, -0.96  -0.39, -0.25  0.005, 0.20  0.13, 0.33 | -0.66 (0.08)  -0.16 (0.06)  -0.02 (0.07)  0.02 (0.09) | -0.81, -0.51  -0.26, -0.06  -0.15, 0.11  -0.16, 0.20 | -0.87, -0.44  -0.30, -0.02  -  -0.21, 0.16  -0.23, 0.28 |
| - Interactions   T2DMxT1  T2DMxT2  T2DMxT3  T2DMxT4  T2DMxT5   - Wald test for interaction term | 0.04 (0.05)  0.01 (0.06)  Reference   - 1. (0.07)   0.07 (0.07) | -0.07, 0.15  -0.10, 0.12  -0.13, 0.15  -0.08, 0.22  (0.881) | 0.02 (0.06)  -0.01 (0.05)  0.02 (0.08)  -0.02 (0.09) | -0.09, 0,14  -0.12, 0.10  -0.13, 0.17  -0.21, 0.16  (0.964) | -0.14, 0.19  -0.16, 0.14  -0.19, 0.23  -0.29, 0.24 |
| - Sex - Age - BMIcat   1  2   - Weight change - Parity - Breastfeeding - Physical activity - Education |  |  | 0.11 (0.09)  0.02 (0.003)  0.04 (0.003)  0.06 (0.06)  0.001 (0.002)  -0.04 (0.03)  0.005 (0.002)  0.04 (0.03)  -0.02 (0.01) | -0.08, 0.29  0.01, 0.02  -0.04, 0.11  -0.05, 0.17  -0.006, 0.005  -0.09, 0.01  -0.000, 0.01  -0.02, 0.10  -0.05, 0.01 | -0.16, 0.37  0.01, 0.03  -0.07, 0.15  -0.10, 0.22  -0.007, 0.007  -0.11, 0.03  -0.002, 0.01  -0.04, 0.13  -0.06, 0.03 |
| - constant | -0.75 (0.04) | -0.84, -0.66 | -1.07 (0.10) | -1.28, -0.87 | -1.37, -0.78 |
| **PFUnDA**   - T2DM | 0.02 (0.08) | -0.13, 0.18 | -0.02 (0.07) | -0.17, 0.12 | -0.24, 0.18 |
| - Sampling year   T1  T2  T3  T4  T5 | -0.33 (0.04)  -0.33 (0.04)  Reference  -0.002 (0.05)  0.01 (0.05) | -0.40, -0.26  -0.41, -0.26  -0.10, 0.10  -0.09, 0.11 | 0.09 (0.08)  -0.13 (0.05)  -0.17 (0.06)  -0.24 (0.09) | -0.06, 0.24  -0.23, -0.03  -0.30, -0.05  -0.42, -0.06 | -0.12, 0.32  -0.26, 0.01  -  -0.35, 0.004  -0.50, 0.01 |
| - Interactions   T2DMxT1  T2DMxT2  T2DMxT3  T2DMxT4  T2DMxT5   - Wald test for interaction term | 0.03 (0.05)  -0.02 (0.05)  Reference  0.03 (0.07)  0.07 (0.07) | -0.08, 0.14  -0.12, 0.09  -0.11, 0.17  -0.07, 0.21  (0.775) | 0.001 (0.06)  -0.04 (0.05)  0.08 (0.07)  -0.06 (0.09) | -0.10, 0.12  -0.14, 0.06  -0.05, 0.23  -0.24, 0.11  (0.327) | -0.15, 0.17  -0.19, 0.10  -0.12, 0.28  -0.31, 0.18 |
| - Sex - Age - Weight change - Parity - Breastfeeding - Physical activity - Education |  |  | 0.18 (0.11)  0.02 (0.003)  -0.001 (0.002)  -0.04 (0.03)  0.004 (0.002)  0.04 (0.03)  -0.03 (0.02) | -0.03, 0.39  0.02, 0.03  -0.01, 0.004  -0.09, 0.02  -0.001, 0.01  -0.02, 0.10  -0.06, 0.002 | -0.12, 0.48  0.01, 0.03  -0.01, 0.005  -0.11, 0.04  -0.003, 0.01  -0.04, 0.12  -0.07, 0.12 |
| - constant | -0.38 (0.05) | -0.49, -0.28 | -0.75 (0.11) | -0.97, -0.53 | -1.10, -0.46 |
| **PFDoDA**   - T2DM | -0.07 (0.10) | -0.27, 0.13 | -0.10 (0.10) | -0.31, 0.10 | -0.40, 0.19 |
| - Sampling year   T1  T2  T3  T4  T5 | -0.75 (0.08)  -0.32 (0.08)  Reference  -0.07 (0.10)  0.03 (0.10) | -0.90, -0.60  -0.47, -0.17  -0.26, 0.13  -0.17, 0.23 | -0.30 (0.13)  -0.13 (0.10)  -0.16 (0.13)  -0.24 (0.16) | -0.56, -0.04  -0.32, 0.07  -0.41, 0.10  -0.56, 0.08 | -0.67, 0.08  -0.41, 0.15  -  -0.52, 0.21  -0.70, 0.22 |
| - Interactions   T2DMxT1  T2DMxT2  T2DMxT3  T2DMxT4  T2DMxT5   - Wald test for interaction term | -0.06 (0.11)  0.02 (0.11)  Reference  0.11 (0.15)  0.04 (0.15) | -0.28, 0.16  -0.20, 0.02  -0.17, 0.40  -0.25, 0.33  (0.833) | -0.03 (0.12)  0.01 (0.11)  0.16 (0.15)  -0.10 (0.18) | -0.26, 0.21  -0.21, 0.23  -0.14, 0.46  -0.44, 0.25  (0.730) | -0.36, 0.31  -0.31, 0.33  -0.27, 0.59  -0.59, 0.40 |
| - Sex - Age - BMIcat   1  2   - Weight change - Parity - Breastfeeding - Physical activity - Education |  |  | 0.09 (0.14)  0.02 (0.004)  0.002 (0.07)  -0.04 (0.10)  0.003 (0.005)  -0.08 (0.04)  0.01 (0.04)  0.09 (0.06)  -0.04 (0.03) | -0.18, 0.37  0.01, 0.03  -0,14, 0,14  -0.23, 0.15  -0.01, 0.01  -0.16, -0.01  0.001, 0.02  -0.02, 0.20  -0.09, 0.01 | -0.30, 0.48  0.01, 0.04  -0.19, 0.20  -0.31, 0.23  -0.01, 0.02  -0.19, 0.02  -0.003, 0.02  -0.07, 0.25  -0.11, 0.04 |
| - Constant | -2.69 (0.07) | -2.82, -2.55 | -3.01 (0.16) | -3.33, -2.69 | -3.47, -2.55 |
| **PFTrDA**   - T2DM | -0.05 (0.10) | -0.24, 0.15 | -0.06 (0.10) | -0.26, 0.15 | -0.34, 0.23 |
| - Sampling year   T1  T2  T3  T4  T5 | -0.35 (0.07)  -0.65 (0.07)  Reference  -0.07 (0.09)  -0.04 (0.09) | -0.48, -0.21  -0.79, -0.52  -0.25, 0.11  -0.23, 0.14 | 0.14 (0.13)  -0.35 (0.09)  -0.01 (0.12)  -0.11 (0.16) | -0.10, 0.39  -0.53, -0.18  -0.25, 0.22  -0.42, 0.19 | -0.21, 0.50  -0.61, -0.10  -  -0.35, 0.32  -0.55, 0.33 |
| - Interactions   T2DMxT1  T2DMxT2  T2DMxT3  T2DMxT4  T2DMxT5   - Wald test for interaction term | -0.21 (0.10)  0.04 (0.10)  Reference  -0.05 (0.13)  -0.10 (0.14) | -0.41, -0.003  -0.16, 0.25  -0.32,0.21  -0.37, 0.18  (0.152) | -0.22 (0.11)  0.01 (0.10)  -0.09 (0.14)  -0.28 (0.17) | -0.43, -0.004  -0.19, 0.21  -0.37, 0.18  -0.61, 0.05  (0.068) | -0.52, 0.09  -0.28, 0.29  -0.48, 0.30  -0.75, 0.19 |
| - Sex - Age - BMIcat   1  2   - Weight change - Parity - Breastfeeding - Physical activity - Education |  |  | 0.35 (0.14)  0.02 (0.004)  0.03 (0.06)  0.003 (0.09)  -0.004 (0.004)  -0.06 (0.04)  0.005 (0.004)  0.08 (0.05)  -0.05 (0.02) | 0.08, 0.62  0.01, 0.03  -0.10, 0.15  -0.17, 0.18  -0.01, 0.006  -0.13, 0.01  -0.003, 0.013  -0.02, 0.18  -0.09, -0.002 | -0.04, 0.73  0.006, 0.03  -0.15, 0.21  -0.25, 0.26  -0.02, 0.01  -0.16, 0.04  -0.007, 0.02  -0.07, 0.23  -0.12, 0.02 |
| - Constant | -2.11 (0.07) | -2.24, -1.98 | -2.49 (0.16) | -2.80, -2.18 | -2-93, -2.06 |
| **PFHxS**   - T2DM | 0.13 (0.07) | -0.01, 0.27 | 0.14 (0.07) | 0.004, 0.27 | -0.05, 0.33 |
| - Sampling year   T1  T2  T3  T4  T5 | -0.88 (0.04)  -0.28 (0.04)  Reference  -0.03 (0.06)  -0.17 (0.06) | -0.97, -0.80  -0.36, -0.19  -  -0.14, 0.08  -0.28, -0.05 | -0.76 (0.08)  -0.22 (0.05)  -  -0.03 (0.07)  -0.12 (0.10) | -0.92, -0.61  -0.33, -0.12  -  -0.16, 0.11  -0.32, 0.07 | -0.98, -0.54  -0.37, -0.08  -  -0.28, 0.17  -0.40, 0.19 |
| - Interactions   T2DMxT1  T2DMxT2  T2DMxT3  T2DMxT4  T2DMxT5   - Wald test for interaction term | -0.10 (0.06)  -0.06 (0.06)  Reference  -0.04 (0.08)  0.04 (0.09) | -0.22, 0.03  -0.19, 0.06  -0.21, 0.12  -0.13, 0.21  (0.423) | -0.17 (0.07)  -0.09 (0.06)  -0.05 (0.08)  -0.10 (0.11) | -0.30, -0.03  -0.20, 0.02  -0.21, 0.11  -0.31, 0.10  (0.132) | -0.35, 0.02  -0.25, 0.07  -0.28, 0.17  -0.40, 0.19 |
| - Sex - Age - BMIcat   1  2   - Weight change - Parity - Breastfeeding - Physical activity - Education |  |  | 0.36 (0.09)  0.01 (0.003)  0.05 (0.04)  0.07 (0.06)  -0.003 (0.003)  0.01 (0.02)  0.002 (0.03)  0.05 (0.03)  -0.01 (0.15) | 0.18, 0.55  0.003, 0.01  -0.03, 0.13  -0.04, 0.18  -0.01, 0.002  -0.04, 0.05  -0.003, 0.01  -0.01, 0.11  -0.04, 0.02 | 0.10, 0.62  0.001, 0.02  -0.06, 0.16  -0.09, 0.23  -0.01, 0.004  -0.06, 0.07  -0.005, 0.01  -0.03, 0.14  -0.05, 0.04 |
| - Constant | 0.63 (0.05) |  | 0.23 (0.10) | 0.03, 0.43 | -0.06, 0.52 |
| **PFHpS**   - T2DM | 0.12 (0.07) | -0.004, 0.25 | 0.06 (0.06) | -0.06, 0.18 | -0.11, 0.24 |
| - Sampling year   T1  T2  T3  T4  T5 | -0.82 (0.04)  -0.29 (0.04)  Reference  -0.19 (0.05)  -0.47 (0.05) | -0.89, -0.75  -0.36, -0.44  -0.29, -0.10  -0.56, -0.37 | -0.66 (0.07)  -0.26 (0.05)  -0.26 (0.06)  -0.57 (0.09) | -0.80, -0.52  -0.34, -0.17  -0.38, -0.15  -0.74, -0.40 | -0.86, -0.46  -0.38, -0.13  -0.43, -0.09  -0.81, -0.32 |
| - Interactions   T2DMxT1  T2DMxT2  T2DMxT3  T2DMxT4  T2DMxT5   - Wald test for interaction term | -0.08 (0.05)  -0.07 (0.05)  Reference  0.02 (0.07)  0.04 (0.07) | -0.18, 0.03  -0.17, 0.04  -0.12, 0.15  -0.10, 0.18  (0.272) | -0.09 (0.06)  -0.07 (0.05)  0.01 (0.07)  -0.03 (0.09) | -0.20. 0.02  -0.17, 0.03  -0.13, 0.14  -0.21, 0.14  (0.506) | -0.25, 0.07  -0.21, 0.07  -0.19, 0.20  -0.28, 0.22 |
| - Sex - Age - BMIcat   1  2   - Weight change - Parity - Breastfeeding - Physical activity - Education |  |  | 0.31 (0.09)  0.01 (0.003)  0.07 (0.04)  0.13 (0.05)  -0.003 (0.002)  0.001 (0.02)  0.003 (0.002)  0.04 (0.03)  -0.002 (0.01) | 0.14, 0.48  0.01, 0.02  -0.002, 0.14  0.03, 0.23  -0.01, 0.002  -0.04, 0.05  -0.001. 0.008  -0.01, 0.09  -0.03, 0.03 | 0.07, 0.55  0.004, 0.02  -0.03, 0.17  -0.01, 0.28  -0.01, 0.004  -0.06, 0.07  -0.003, 0.01  -0.04, 0.12  -0.04, 0.04 |
| - Constant | -0.59 (0.04) | -0.67, -0.50 | -1,01 (0.09) | -1.20, -0.82 | -1.28, -0.74 |
| **PFOS**   - T2DM | 0.06 (0.06) | -0.06, 0.18 | 0.01 (0.06) | -0.11, 0.13 | -0.16, 0.17 |
| - Sampling year   T1  T2  T3  T4  T5 | -0.68 (0.03)  -0.22 (0.03)  Reference  -0.44 (0.05)  -0.90 (0.05) | -0.75, -0.61  -0.29, -0.16  -0.53, -0.35  -0.99, -0.80 | -0.50 (0.07)  -0.18 (0.05)  -0.53 (0.06)  -1.02 (0.08) | -0.64, -0.37  -0.27, -0.10  -0.65, -0.42  -1.18, -0.86 | -0.70, -0.31  -0.31, -0.06  -  -0.70, -0.36  -1.25, -0.78 |
| - Interactions   T2DMxT1  T2DMxT2  T2DMxT3  T2DMxT4  T2DMxT5   - Wald test for interaction term | -0.04 (0.05)  -0.08 (0.05)  Reference  0.07 (0.07)  0.17 (0.07) | -0.14, 0.06  -0.18, 0.02  -0.06, 0.20  0.03, 0.30  (0.004) | -0.05 (0.05)  -0.08 (0.05)  0.07 (0.07)  0.12 (0.09) | -0.16, 0.06  -0.18, 0.01  -0.06, 0.21  -0.05, 0.28  (0.154) | -0.21, 0.10  -0.22, 0.05  -0.12, 0.26  -0.12, 0.36 |
| - Sex - Age - BMIcat   1  2   - Weight change - Parity - Breastfeeding - Physical activity - Education |  |  | 0.26 (0.08)  0.01 (0.003)  0.05 (0.03)  0.11 (0.05)  0.001 (0.002)  0.004 (0.02)  0.002 (0.002)  0.04 (0.03)  0.006 (0.01) | 0.10, 0.43  0.01, 0.02  -0.02, 0.12  0.01, 0.21  -0.005, 0.003  -0.04, 0.05  -0.002, 0.01  -0.01, 0.09  -0.02, 0.03 | 0.03, 0.50  0.003, 0.02  -0.05, 0.14  -0.03, 0.25  -0.01, 0.005  -0.06, 0.06  -0.004, 0.01  -0.04, 0,11  -0.03, 0.04 |
| - Constant | 3.59 (0.04) | 3.51, 3.68 | 3.21 (0.09) | 3.03, 3.39 | 2.95, 3.47 |
| **PFOSA**   - T2DM | 0.18 (0.10) | -0.03, 0.38 | 0.17 (0.11) | -0.04, 0.39 | -0.14, 0.49 |
| - Sampling year   T1  T2  T3  T4  T5 | 0.04 (0,09)  0.26 (0.09)  Reference  -2.13 (0.12)  -2.18 (0.12) | -0.14, 0.21  0.08, 0.44  -2.36, -1.90  -2.41, -1.94 | 0.36 (0.15)  0.38 (0.12)  -2.25 (0.16)  -2.40 (0.19) | 0.07, 0.66  0.15, 0.61  -2.55, -1.94  -2.78, -2.03 | -0.06, 0.79  0.04, 0.72  -  -2.68, -1.81  -2.94, -1.87 |
| - Interactions   T2DMxT1  T2DMxT2  T2DMxT3  T2DMxT4  T2DMxT5   - Wald test for interaction term | -0.44 (0.13)  -0.21 (0.13)  Reference  0.03 (0.17)  -0.41 (0.18) | -0.71, -0.18  -0.47, 0.06  -0.30, 0.37  -0.76, -0.06  (0.004) | -0.52 (0.14)  -0.21 (0.14)  -0.03 (0.18)  -0.43 (0.21) | -0.80, -0.24  -0.49, 0.06  -0.40, 0.33  -0.84, -0.01  (0.003) | -0.91, -0.12  -0.61, 0.18  -0.55, 0.49  -1.02, 0.17 |
| - Sex - Age - BMIcat   1  2   - Weight change - Parity - Breastfeeding - Physical activity - Education |  |  | 0.27 (0.14)  0.01 (0.004)  0.003 (0.08)  0.005 (0.10)  -0.01 (0.006)  0.02 (0.04)  0.003 (0.005)  0.002 (0.06)  0.01 (0.03) | -0.003, 0.54  0.01, 0.02  -0.15, 0.15  -0.19, 0.20  -0.02, 0.004  -0.05, 0.09  -0.01, 0.01  -0.13, 0.13  -0.04, 0.06 | -0.12, 0.66  0.003, 0.03  -0.21, 0.22  -0.27, 0.28  -0.02, 0.01  -0.08, 0.12  -0.01, 0.02  -0,18, 0.18  -0.07, 0.08 |
| - Constant | -3.71 (0.07) | -3.85, -3.57 | -4.11 (0.16) | -4.43, -3.79 | -4.57, -3.65 |

**Table S6. Reported intake of fatty and lean fish presented as means and standard deviations (SD) for cases and controls, and the mean difference (Δ) between type 2 diabetes mellitus cases and controls at each pre- and post-diagnostic time-points (T). The Tromsø study (1986-2016)**

|  |  | **Pre-diagnostic time-points** | | | | | | | **Post-diagnostic time-points** | | | |
| --- | --- | --- | --- | --- | --- | --- | --- | --- | --- | --- | --- | --- |
| **Characteristics** |  | **T1 (1986/87)** | | **T2 (1994/95)** | | **T3 (2001)** | | | **T4 (2007/08)** | | **T5 (2015/16)** | |
|  |  | **Mean±SD** | **ΔMean**  **(95% CI)** | **Mean±SD** | **ΔMean**  **(95% CI)** | **Mean±SD** | **ΔMean**  **(95% CI)** | **Mean±SD** | | **ΔMean**  **(95% CI)** | **Mean±SD** | **ΔMean**  **(95% CI)** |
| **Fatty fish consumption^f^** | Cases  Controls | 1.24 (0.05)  1.20 (0.04) | 0.06  (-0.05, 0.22) | 2.62 (0.09)  2.50 (0.08) | 0.12  (-0.11, 0.36) | 2.42 (0.08)  2.33 (0.07) | 0.10 (-0.12, 0.32) | 2.41 (0.09)  2.39 (0.09) | | 0.02  (-0.24, 0.28) | 2.48 (0.13)  2.54 (0.10) | -0.06  (-0.36, 0.25) |
| **Lean fish consumption^g^** | Cases  Controls | 2.77 (0.09)  2.50 (0.08) | 0.27  (0.03, 0.51) | 3.44 (0.08)  3.28 (0.07) | 0.17  (-0.04, 0.38) | NA  NA | - | 2.99 (0.08)  2.93 (0.08) | | 0.05  (-0.17, 0.28) | 2.98 (0.08)  2.83 (0.09) | 0.15  (-0.09, 0.39) |

T1: n=255, 116 cases; T2: n=252, 115 cases; T3: n=255, 116 cases; T4: n=120, 57 cases; T5: n=108, 50 cases, NA: Not available

^f^Self reported intake with frequency in increasing order with the alternatives 1-4 (T1) or 1-6 (T2), 1-5 (T4 and T5).

**
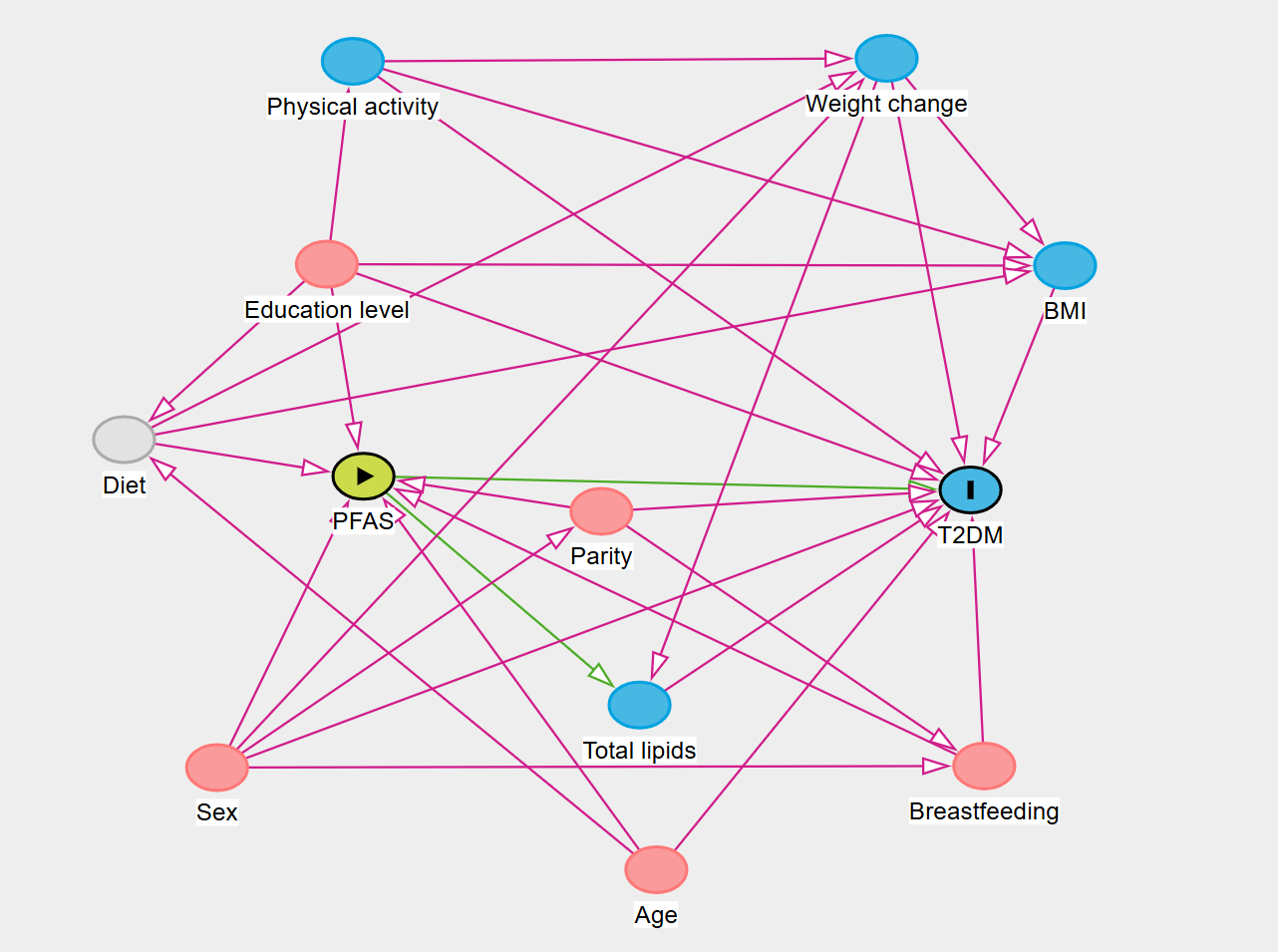
**

**Figure S1. The causal relationship between perfluoro alkyl substances (PFAS) and type 2 diabetes mellitus (T2DM) and potential confounders, illustrated in a directed acyclic graph (DAG). The different colors represent; green-exposure; blue-outcome; pink-confounders; grey-unobserved; arrows-direction of the pathways.**

According to the DAG, the sufficient adjustment set for estimating the total effect of PFAS on T2DM includes adjusting for age, breastfeeding, parity, physical activity, sex, BMI, weight change, and education level. The factors (in pink) are considered confounders as they directly or indirectly, through mediators (which are also confounders), affect both PFAS concentrations and T2DM. Therefore, they should be adjusted for in data analyses. Age is an established risk factor for T2DM and an important determinant of blood PFAS concentrations. In women, breastfeeding and parity directly influence PFAS concentrations, and low breastfeeding rates and increased parity have been shown to elevate the risk of T2DM. Furthermore, sex directly influences PFAS concentrations and may increase the risk of T2DM through its effects on breastfeeding and/or parity. Educational level reflects socioeconomic status (SES), which is associated with the risk of T2DM and is also linked to PFAS concentrations and physical activity. Diet directly influences PFAS concentrations (through the consumption of foods containing PFAS); however, its effect on T2DM may be mediated through BMI and weight change. Total lipids are potential mediators, as PFAS may cause increased lipid levels, which are associated with the risk of T2DM. Lipids are also linked to weight change. Low physical activity is a known risk factor for T2DM and may influence PFAS concentrations through weight changes associated with physical activity. Dietary variables were poorly recorded during the early time-points (T1-T3) and were therefore set as unobserved.
